# Coordinated repression of BIM and PUMA by Epstein–Barr virus latent genes maintains the survival of Burkitt lymphoma cells

**DOI:** 10.1038/cdd.2017.150

**Published:** 2017-09-29

**Authors:** Leah Fitzsimmons, Andrew J Boyce, Wenbin Wei, Catherine Chang, Deborah Croom-Carter, Rosemary J Tierney, Marco J Herold, Andrew I Bell, Andreas Strasser, Gemma L Kelly, Martin Rowe

**Affiliations:** 1 Institute of Cancer and Genomic Sciences and Centre for Human Virology, University of Birmingham, College of Medical and Dental Sciences, Birmingham B15 2TT, UK; 2 Sheffield Institute of Translational Neuroscience, University of Sheffield, Sheffield, UK; 3 The Walter and Eliza Hall Institute for Medical Research, Parkville, VIC 3052, Australia; 4 Department of Medical Biology, The University of Melbourne, Parkville, VIC 3052, Australia

## Abstract

While the association of Epstein–Barr virus (EBV) with Burkitt lymphoma (BL) has long been recognised, the precise role of the virus in BL pathogenesis is not fully resolved. EBV can be lost spontaneously from some BL cell lines, and these EBV-loss lymphoma cells reportedly have a survival disadvantage. Here we have generated an extensive panel of EBV-loss clones from multiple BL backgrounds and examined their phenotype comparing them to their isogenic EBV-positive counterparts. We report that, while loss of EBV from BL cells is rare, it is consistently associated with an enhanced predisposition to undergo apoptosis and reduced tumorigenicity *in vivo*. Importantly, reinfection of EBV-loss clones with EBV, but surprisingly not transduction with individual BL-associated latent viral genes, restored protection from apoptosis. Expression profiling and functional analysis of apoptosis-related proteins and transcripts in BL cells revealed that EBV inhibits the upregulation of the proapoptotic BH3-only proteins, BIM and PUMA. We conclude that latent EBV genes cooperatively enhance the survival of BL cells by suppression of the intrinsic apoptosis pathway signalling via inhibition of the potent apoptosis initiators, BIM and PUMA.

Epstein–Barr virus (EBV)-positive Burkitt lymphoma (BL), an aggressive and difficult to treat malignancy, is endemic (eBL) in sub-Saharan Africa, where it accounts for around half of all childhood lymphomas. BL also occurs worldwide at lower incidence, and in these cases, known as sporadic BLs (spBL), EBV is found in 15–85% of tumours, varying by geographical region.^1^ The genetic hallmark of all BL is the chromosomal translocation between the c-*MYC* gene and a constitutively active immunoglobulin (Ig) gene promoter/enhancer. Typical of *c-MYC*-driven lymphomas, BLs proliferate rapidly but are also sensitive to apoptosis under conditions of stress.^2^

Cellular proapoptotic BH3-only proteins (e.g. BIM, PUMA, BAD, NOXA) induce cell death by unleashing the proapoptotic multi-BH domain executioner BCL-2 family members, BAK and BAX, allowing them to form pores in the mitochondrial outer membrane, which commits the cells to apoptosis. BH3-only proteins can achieve this by binding and inhibiting prosurvival BCL-2 family proteins (BCL-2, BCL-XL, BCL-W, A1/BFL1 and MCL-1), which in the absence of an apoptotic stimulus restrain BAK and BAX, thereby preventing cellular destruction. Additionally, BIM, PUMA and tBID can directly activate BAX and BAK (reviewed in Strasser *et al.*^3^). It was shown using the *Eμ-Myc* transgenic mouse model of human BL (which expresses a *c-Myc;Ig* transgene) that blocking apoptosis through enforced expression of BCL-2 prosurvival proteins or deletion of BH3-only proteins or BAX greatly accelerates lymphoma development.^4, 5, 6, 7^

In human BL it is not clear how EBV contributes to the continued growth of the tumour. One view is that EBV counteracts the cell death-promoting actions of aberrant *c-MYC* expression. When EBV infects resting B cells *in vitro*, it expresses the growth-transforming programme of latency genes, termed Latency III, involving expression of 10 proteins (EBNAs 1, 2, 3A, 3B, 3C and LP; LMPs 1, 2A and 2B; and the viral BCL-2 homologue, BHRF1), two non-coding RNAs (EBER1 and EBER2), and two families of microRNAs (BART microRNAs (miR-BARTs) and miR-BHRF1s), which together drive proliferation and promote cell survival (reviewed in Rowe *et al.*^8^) (Figure 1).

Crucially, however, most of the Latency III genes are not expressed in established BLs. Instead, in BLs, EBV exhibits more restricted forms of latency characterised by expression of EBNA1, the EBER transcripts and the miR-BARTs (Figure 1). Only a minority of eBLs exhibit a more complex viral gene expression pattern due to a genomic deletion in EBV.^9, 10^ Cell lines derived from these tumours show marked resistance to apoptosis due to epigenetic silencing of the *BIM* promoter^11^ and functional inhibition of BIM, PUMA, BID and BAK by the viral BCL-2 homologue, BHRF1.^12^

EBV-positive and -negative BLs are genetically distinct, differing in terms of their cellular mutational profiles^13, 14^ and precise *Ig-MYC* chromosomal translocations.^15, 16^ It is therefore unsatisfactory to introduce the virus or viral genes into EBV-negative spBL lines to study the role of EBV in eBL. Instead, efforts have focused on trying to rid EBV-positive eBLs of the virus to assess the contribution of EBV to the growth and survival of BL in an isogenic system. Treatment of EBV-positive BL cells with a dominant-negative form of EBNA1 leads to loss of EBV genomes and widespread apoptosis.^17, 18, 19, 20^ While implying that EBV is essential for the continued survival of BL cells, this method yielded few EBV-loss clones for mechanistic studies. Hydroxyurea treatment can also eradicate EBV, but these BL clones do not show a consistent apoptosis predisposition phenotype.^21^ Additionally, an unusual EBV-positive spBL (Akata-BL) cell line has been reported to spontaneously lose EBV *in vitro*, yielding clones with impaired cell growth.^22^

A significant limitation of these previous studies has been the small number of tumour backgrounds and/or clones analysed. BL, like many cancers, can exhibit considerable inter- and intratumoral genetic heterogeneity.^23, 24^ The present study resolves the unmet need for a systematic approach to analyse multiple EBV-loss BL clones on several tumour backgrounds both *in vitro* and *in vivo* in a xenograft model of BL. This work has shown unequivocally that EBV in a Latency I infection can protect BL cells from apoptosis mediated by the proapoptotic BH3-only proteins, BIM and PUMA.

## Results

### Determining the contribution of EBV to the continued growth of BL cells

**Figure 2:**
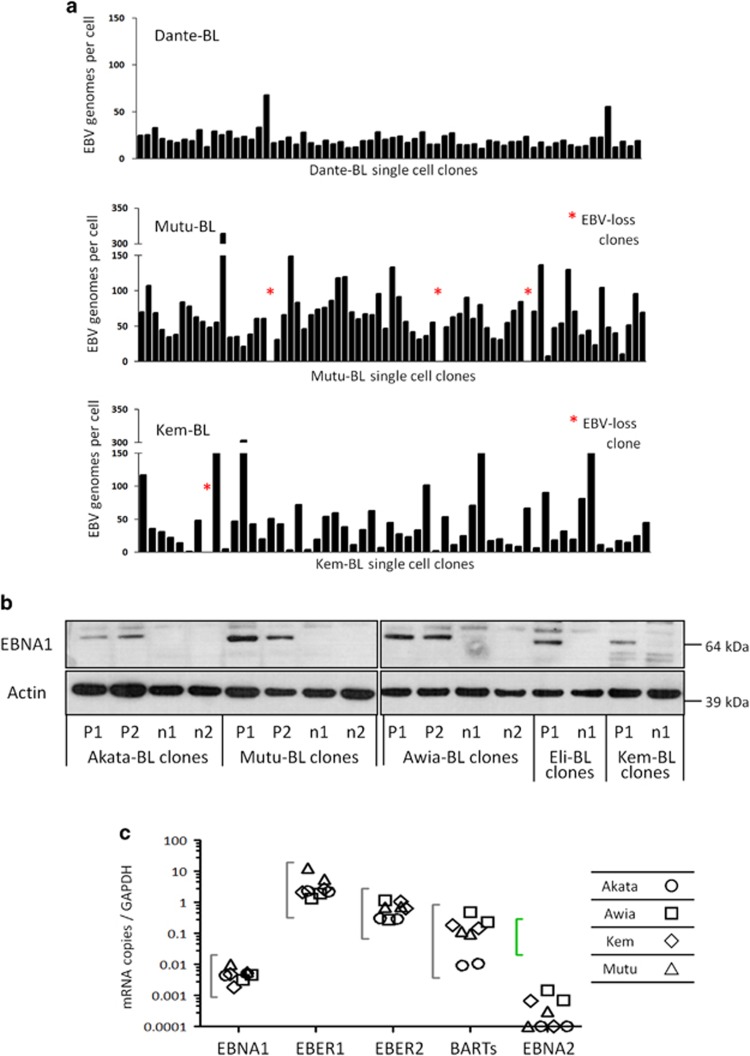
Genome loads and viral gene expression in BL single-cell clones. (**a**) Cells from each clonal cell line grown from a single cell by limiting dilution were harvested, lysed and analysed by q-PCR to enumerate the average EBV genome copy number per cell. Quantitation was calculated relative to Namalwa-BL cells, which contain two integrated copies of EBV per cell and these data were normalised to the housekeeping gene, *β2M*, of which diploid cells carry two copies. Clones of Dante-BL showed little variation in EBV copy number and yielded no EBV-loss clones, whereas Mutu-BL and Kem-BL cells had more variable genome loads and yielded EBV-loss clones (denoted by an asterisk). (**b**) Expression of EBV latent protein, EBNA1, in isogenic EBV-positive (P1-P2) and EBV-loss (n1-n2) BL clones. Probing for *β*-actin was used as a loading control. (**c**) Transcription of Latency I-associated genes in EBV-positive clones of Akata-BL (◯), Awia-BL (□), Kem-BL (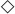) and Mutu-BL (Δ) expressed relative to the endogenous control, GAPDH, and shown relative to the range seen in a panel of eight Latency I BL cell lines (grey bracket), including those from which the EBV-loss clones were isolated, as described elsewhere.^53^ EBNA2 expression is shown compared with the range seen in a panel of five LCLs (green bracket). EBNA1 refers to Q-U-K transcripts driven from the Qp promoter that are indicative of Latency I. BARTs refers to *Bam*HI A transcripts that are spliced between exons 1 and 3 (the excised RNA gives rise to miR-BARTs)

A panel of EBV-positive BL cell lines (1 spBL and 11 eBLs) were seeded at single-cell dilutions to establish more than 1800 clones. These clones were screened for EBV episome copy number by quantitative, real-time PCR (q-PCR); the results are summarised in Table 1 and Figure 2a. Strikingly, the generation of EBV-loss cells was a rare event, observed in only 61/1800 (3.4%) clones and seven BL cell lines never yielded EBV-loss clones. These results strongly indicate that while EBV is not essential for continued BL cell growth, there is strong selective pressure to retain EBV.

Before investigating the phenotype of the EBV-positive clones, their EBV infection status was fully characterised. This included flow cytometry for EBERs to confirm that all cells carried the virus, as well as immunoblotting and q-PCR for 45 EBV transcripts to ensure that the clones retained Latency I gene expression (EBNA1-positive, EBNA2- and LMP1-negative, and low lytic cycle gene expression) (Figures 2b and c and Supplementary Figure 1).

### Effect of EBV loss on the tumorigenicity and survival phenotype of BL clones

**Figure 3:**
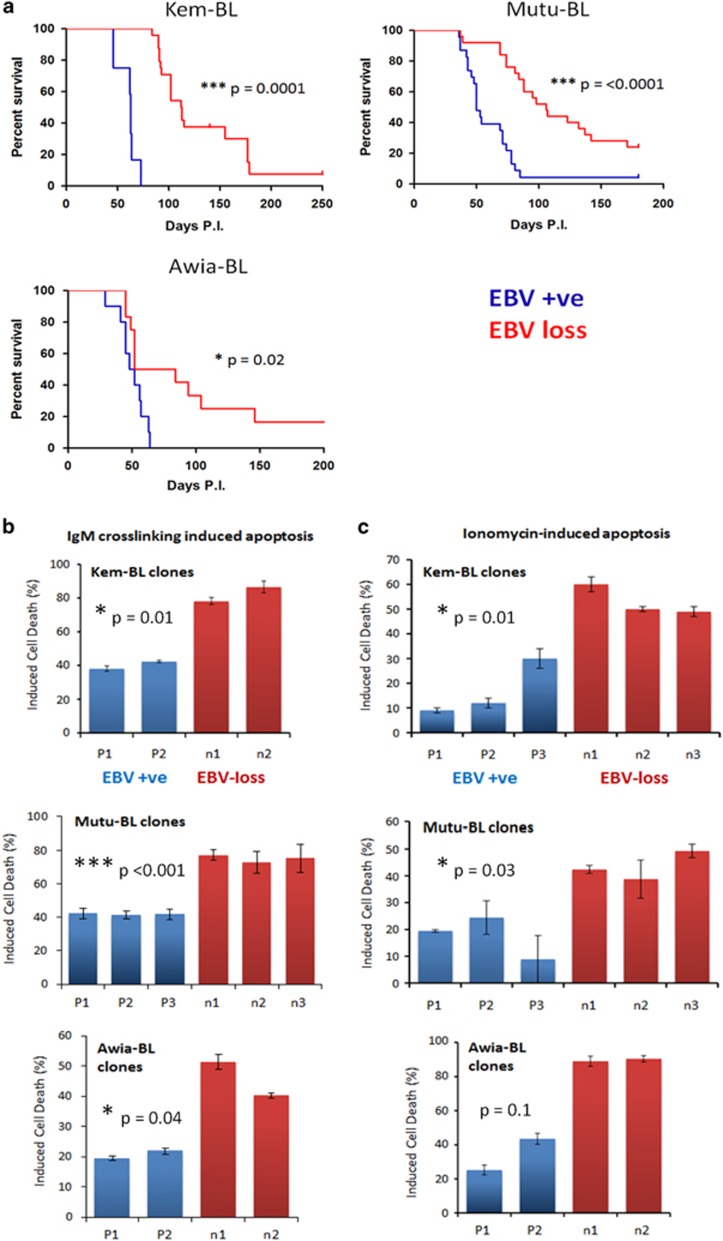
*In vivo* and *in vitro* phenotype of isogenic EBV-positive and EBV-loss BL clones. (**a**) Tumorigenicity of BL clones in NSG mice. Kaplan–Meier survival plots comparing survival in days post inoculation (P.I.) with isogenic EBV-positive (blue) or EBV-loss clones (red) derived from the parental EBV-positive BL cells lines as indicated. Kem-BL clones, EBV-positive *n*=12, EBV-loss *n*=24, median survival (MS) 54*versus* 102 days. Mutu-BL clones, EBV-positive *n*=19, EBV-loss *n*=29, MS 63*versus* 113 days. Awia-BL clones, EBV-positive *n*=12, EBV-loss *n*=12, MS 50*versus* 68 days. (**b**) Apoptotic cell death induced in response to BCR crosslinking with anti-IgM antibodies in isogenic EBV-positive (P1–P3) *versus* EBV-loss (n1–n3) clones of Kem-BL, Mutu-BL and Awia-BL, 72 h post-treatment compared with vehicle-only controls. (**c**) Ionomycin-induced cell death in isogenic EBV-positive (P1–P3) *versus* EBV-loss (n1–n3) clones of Kem-BL, Mutu-BL and Awia-BL (48 h), compared with vehicle-treated controls. Data are the mean and standard deviation (S.D.) of pooled data from three independent experiments, each carried out in triplicate. Unpaired, two-tailed Student’s *T*-tests were carried out to assess the significance of any difference in response between EBV-positive and EBV-loss clones from each background and *P*-values are indicated. Additionally, a two-way analysis of variance (ANOVA) to compare all clones from all BL tumours showed that overall EBV has a highly significant effect on cell survival (*P*<0.0001) in response to both ionomycin and IgM crosslinking

To assess the contribution of EBV to BL growth *in vivo*, isogenic EBV-positive and EBV-loss clones derived from three eBL backgrounds (Kem-BL, Mutu-BL and Awia-BL), were transplanted by intraperitoneal injection into female NSG mice and the animals monitored for tumour burden. Tumours arose in the spleen, ovaries and pancreas and resembled human BL in terms of pathology and histological ‘starry sky’ appearance (Supplementary Figure 2a). Across all tumour backgrounds, EBV-loss clones were significantly less tumorigenic than their EBV-positive counterparts. The median survival of EBV-positive *versus* EBV-loss clones was 54*versus* 102 days for Kem-BL, 63*versus* 113 days for Mutu-BL and 50*versus* 68 days for Awia-BL (Figure 3a). For direct comparison with previous studies,^22, 25^ clones of Akata-BL were transplanted by subcutaneous injection into NSG mice at a higher inoculum. The EBV-positive clones gave rise to tumours, whereas the EBV-negative clones were non-tumorigenic *in vivo* (Supplementary Figure 2b). Subsequent *ex vivo* analysis confirmed that the Latency I gene expression pattern was retained in all tumours derived from EBV-positive BL cells (Supplementary Figure 2c).

To explain the loss of tumorigenic potential *in vivo*, isogenic EBV-positive and EBV-loss clones were treated with apoptosis-inducing agents *in vitro* to ascertain whether EBV confers a survival advantage under stress conditions. EBV-loss clones were consistently more sensitive to apoptosis induced by BCR crosslinking or treatment with ionomycin, roscovitine or staurosporine (Figures 3b and c and Supplementary Figures 2a and 3a, b). Consistent with the finding that the TP53 tumour suppressor pathway is frequently abrogated in human BL,^26^ there was no difference in survival of the EBV-loss and EBV-positive clones following treatment with the DNA damage inducers, etoposide and cisplatin (Supplementary Figure 3c and data not shown). Furthermore, all BL clones were resistant to agents that induce cell death via the extrinsic apoptosis pathway (Supplementary Figure 4), in agreement with published findings.^27^ These data suggest that EBV confers a survival advantage to BL cells through modulation of the intrinsic, BCL-2 family-regulated, apoptotic pathway.

### Effect of Latency I EBV genes on BL cell survival

**Figure 4:**
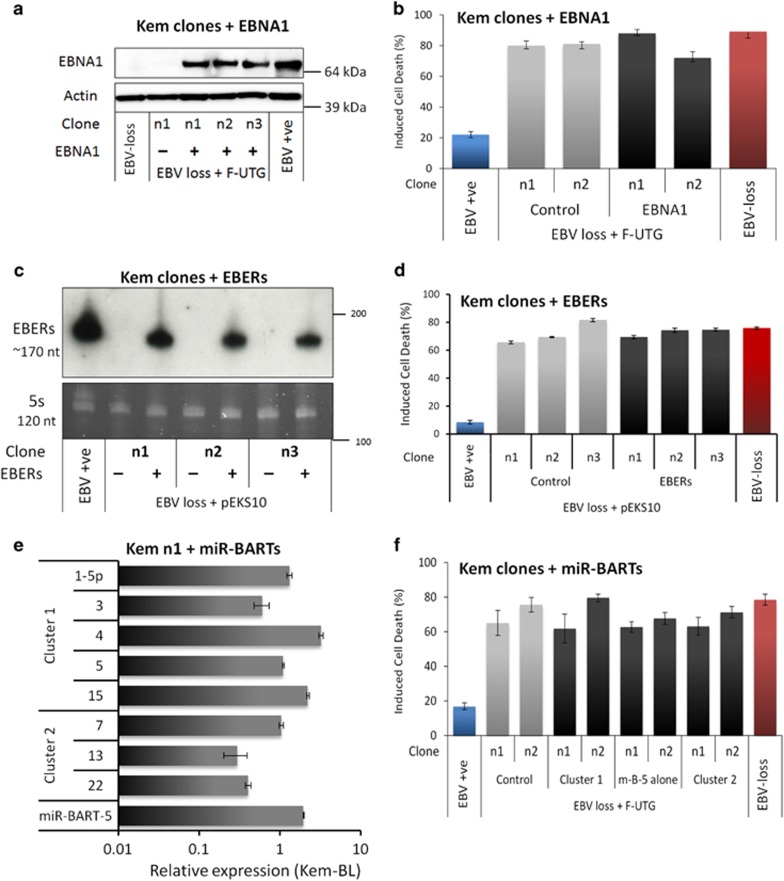
Apoptotic phenotype of EBV-loss Kem-BL clones re-expressing Latency I-associated genes. EBV-loss Kem-BL clones (n1–n3) re-expressing EBNA1 (**a** and **b**), EBERs (**c** and **d**), miR-BARTs (**e** and **f**) or empty vector (controls), marked (+) and (−), respectively, compared with isogenic EBV-loss (EBV −ve) and parental EBV-positive cells (EBV +ve). (**a**) EBNA1 protein expression, blotted with human AMo serum with probing for actin used as a loading control. (**b**) Survival of ionomycin-treated EBNA1-expressing EBV-loss BL cells compared with controls. (**c**) EBER expression was detected by Northern blotting using a probe to the *Eco*RI J fragment of the EBV genome, using 5 S as a loading control. (**d**) Survival of ionomycin-treated, EBER-expressing EBV-loss BL cells compared with controls. (**e**) Expression of mature BART miRs by q-PCR, expressed from the Cluster 1 (top), Cluster 2 (middle) or miR-BART-5 only (bottom) constructs, relative to levels in Kem-BL. (**f**) Survival of ionomycin-treated miR-BART-expressing EBV-loss BL cells compared with controls. All F-UTG-transduced cells in (**a**,**b**,**e** and **f**) induced with dox for 24 h before experiments were carried out. In apoptosis assays (**b**,**d** and **f**), cell death was induced by treatment with ionomycin for 48 h. Data are representative of assays that were carried out in triplicate on three independent occasions

The literature mapping EBV protection to a Latency I gene product (namely EBNA1 protein, EBERs or miR-BARTs), is highly contradictory^18, 25, 28, 29, 30, 31, 32, 33^ (reviewed in the Discussion). To broaden these assorted but limited analyses, each of the Latency I-associated genes were introduced into multiple EBV-loss clones derived from several tumours. Figure 4 depicts data on Kem-BL clones; data from Mutu-BL and Akata-BL clones are shown in Supplementary Figures 5 and 6, respectively. Lentiviral vectors^34^ were used to express EBNA1 protein and the BART miRs in EBV-loss cells at levels equivalent to those detected in EBV-positive BLs (Figures 4a and e and Supplementary Figures 5a, e and 6a, e). A derivative of pcDNA3 (pEKS10), containing 10 tandem repeats of the *Eco*RI J region of the Akata strain EBV genome (KC207813.1),^29^ was used to efficiently express physiologically high levels of EBER RNAs (Figure 4c and Supplementary Figures 5c and 6c).

Perhaps surprisingly, expression of neither EBNA1 nor EBERs conferred any survival or growth advantage to EBV-loss clones across three tumour backgrounds (Figures 4b and d and Supplementary Figures 5b, d and 6b, d). In contrast to published findings, EBER expression also did not upregulate IL-10 and exogenously added IL-10 only modestly increased BL cell survival (Supplementary Figure 7).^35^ Expression of cluster 1 miR-BARTs, cluster 2 miR-BARTs or miR-BART-5 alone also conferred no protection to EBV-loss BL clones (Figure 4f and Supplementary Figures 5f and 6f). A downregulation of PUMA by miR-BART-5 was observed in 293 cells, as reported previously,^36^ but no effect on BIM was apparent^37^ (Supplementary Figure 8a). Critically, this downregulation of PUMA was not observed in EBV-loss BL cells expressing cluster 1 miR-BARTs, cluster 2 miR-BARTs or miR-BART-5 alone (Supplementary Figure 8b). To summarise, no Latency I-associated EBV gene product alone could restore apoptosis protection to EBV-loss BL clones, suggesting that EBNA1, EBERs and miR-BARTs may function cooperatively to inhibit apoptosis in BL.

**Figure 5:**
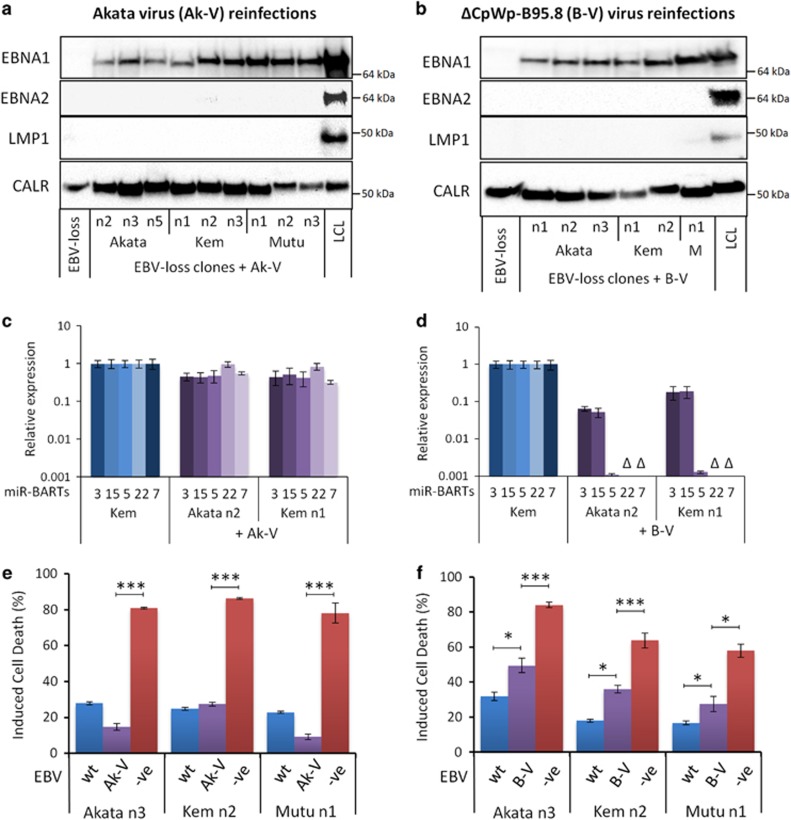
EBV gene expression and apoptosis sensitivity in EBV-loss clones of BL reinfected with Akata virus or ΔCpWp-B95.8 rEBVs. (**a** and **b**) Western blot analysis of EBV latent protein expression in EBV-loss BL clones of Akata (An1-5), Kem (Kn1-3) and Mutu (Mn1-3) reinfected with Akata virus (Ak-V) (panel a, left) or ΔCpWp-B95.8 (B-V) virus (panel b, right). Latency III LCLs express EBNA1, EBNA2 and LMP1, but Latency I reinfectants express only EBNA1 protein. EBV-loss cells that had not been reinfected express only the cellular loading control, calregulin (CALR). (**c** and **d**) Expression of miR-BARTs in EBV-loss clones reinfected with Akata virus (Ak-V) (panel c, left) or ΔCpWp-B95.8 virus (B-V) (panel d, right) quantified using stem-loop real-time PCR. Representative data for three miRNAs from BART cluster 1 (miR-BARTs 3, 15 and 5) and two from BART cluster 2 (miR-BARTs 22 and 7) are shown relative to levels in the EBV-positive, Latency I cell line, Kem-BL. Note: the ΔCpWp-B95.8 virus genome harbours a deletion spanning miR-BARTs 22 and 7 (denoted as Δ) as well as the 3′ end of the miR-BART-5 pre-miRNA. (**e**) Survival of EBV-loss clones reinfected with Akata virus (Ak-V, purple) after challenge with ionomycin for 48 h relative to untreated controls and compared with EBV-positive parental BL cells (wt, blue) and EBV-loss BL cells from the same clonal background (−ve, red). (**f**) Survival of EBV-loss BL clones reinfected with ΔCpWp-B95.8 virus (B-V, purple) after challenge with ionomycin for 48 h relative to untreated controls and compared with EBV-positive parental BL cells (wt, blue) and EBV-loss BL cells from the same clonal background (−ve, red). Data are presented as the mean and S.D. of three independent experiments, each carried out in triplicate. Statistical significance was determined using an unpaired, two-tailed Student’s *T*-test, **P<0.01, *P<0.05

To confirm that apoptosis protection is EBV mediated, we sought to reinfect EBV-loss BL variants with recombinant (r)EBV. These experiments are not trivial as reinfection with EBV usually gives rise to the extensive Latency III infection rather than a restricted Latency I pattern,^38^ and reinfectants often harbour EBV as a small number of integrated copies, rather than as multiple episomes typical of eBL.^39^ Two different recombinant viruses were utilised – a recombinant derivative of the Akata virus, Akata-GFP2 (Ak-V)^40^ and a variant of the B95.8 prototype strain termed ΔCpWp-B95.8 (B-V), which is forced to adopt a Latency I infection because of genomic deletions spanning the Latency III-associated viral promoters, Cp/Wp^41^ (Supplementary Figure 9). Reinfectants were selected for functional analysis if >90% EBV-positive by GFP or EBER-ISH, they contained >10 EBV genomes per cell by DNA q-PCR and exhibited a Latency I infection as determined by q-PCR, IF and immunoblotting (Figures 5a–d and Supplementary Figure 10, and data not shown). A total of nine Ak-V and six B-V reinfected cell lines across the Akata-BL, Kem-BL and Mutu-BL tumour backgrounds met these criteria.

Importantly, Ak-V could protect EBV-loss BL cells from apoptotic stimuli; six cell lines were fully protected, two were substantially protected and one (Akata n2) showed no protection (Figures 5e and f and Supplementary Figure 11 and summarised in Table 2). Reinfection of EBV-loss BL clones with B-V gave some apoptosis protection (except for clone Akata n2), but these clones were significantly more sensitive to apoptosis than the original EBV-positive counterparts. Therefore, EBV in a Latency I infection generally confers apoptosis resistance to BL. The difference in apoptosis protection conferred by the two viruses is intriguing and may be due to one or more genes encoded within a 12 kb region of the EBV *Bam*HI A genomic locus, which is naturally deleted in B95.8 and its derivative, B-V.^42, 43^ These data highlight the clonal variation and emphasises the need to analyse multiple clones before drawing general conclusions.

### Effect of EBV loss on cellular gene expression in BL

To determine which cellular genes are modulated by EBV in Latency I BL, RNA was extracted from EBV-positive and -loss clones from four BL backgrounds (Akata-BL, Eli-BL, Mutu-BL and Awia-BL) and microarray gene expression analysis was undertaken. Surprisingly, no genes were significantly changed more than twofold in EBV-loss compared with EBV-positive clones across all tumours. Additionally, no differences in steady-state expression of BCL-2 family transcripts were observed (Supplementary Figure 12). We were also unable to validate reports that expression levels of c-MYC, miR-127 and -199a or surface immunoglobulin correlate with the presence of EBV (data not shown).^30, 44, 45, 46^

To investigate the possibility that EBV imposes gene expression changes only under conditions of stress, three EBV-positive and EBV-loss clones of Kem-BL were treated with ionomycin. As expected, ionomycin induced more cell death in the EBV-loss clones than the EBV-positive clones (36% *versus* 79% viability), with concordant cleavage of PARP and increased caspase activation (Supplementary Figure 13). In the presence of the pancaspase inhibitor Q-VD.OPh, all ionomycin-treated clones remained >90% viable, permitting RNA to be extracted post-treatment and microfluidic q-PCR cards used to quantify changes in the expression of cell death-associated transcripts.

**Figure 6:**
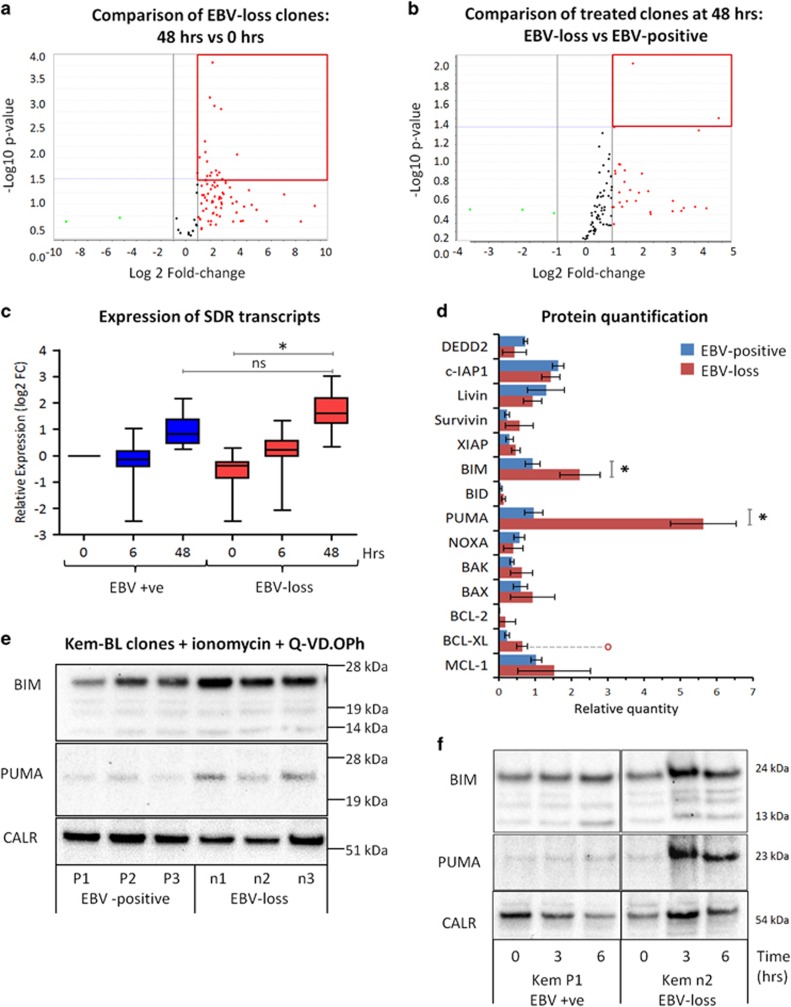
Apoptosis-related transcript and protein expression in ionomycin+Q-VD.OPh-treated Kem-BL clones. (**a**) Volcano plot of changes in apoptosis-related transcripts in EBV-loss BL clones treated with ionomycin and Q-VD.OPh for 48 h *versus* 0 h. The red box indicates significantly differentially regulated genes, as determined using cutoffs of FC >2 and *P*-value <0.05. (**b**) Volcano plot of transcriptional changes in apoptosis-related genes in EBV-loss clones compared with their EBV-positive counterparts, treated with ionomycin and Q-VD.OPh for 48 h. The red box indicates the significantly differentially regulated genes, as determined using cutoffs of FC >2 and *P*-value <0.05. (**c**) Box plot of expression data for the 13 genes that are differentially regulated between EBV-loss clones treated for 48 *versus* 0 h, but not between EBV-positive and EBV-loss BL clones at 48 h. This subset appears to be an EBV-loss-specific gene expression signature. However, this comparison illustrates that this subset of genes is also upregulated in EBV-positive BL clones, but to a lesser extent than in the clones that have lost EBV. (**d**) Summary of apoptosis-related protein expression in EBV-positive (blue) *versus* EBV-loss (red) clones of Kem-BL treated with ionomycin and Q-VD.OPh for 48 h. Quantitation is relative to untreated Jurkat cells. Three proteins, which we found to be undetectable in Kem-BL, are omitted (CFLAR, BAD and CASP8AP2). Data are presented as mean and S.D. of three separate experiments. Red circle in BCL-XL expression data represents one outlier result from a single EBV-loss clone. (**e**) BIM and PUMA protein expression in EBV-positive (P1–P3) *versus* EBV-loss (n1–n3) Kem-BL clones treated with ionomycin and Q-VD.OPh for 48 h. Calregulin (CALR) was included as a loading control. Images are representative from three independent experiments. (**f**) Western blots showing BIM and PUMA expression in EBV-positive Kem-P1 cells compared with EBV-loss Kem-n2 cells at 0, 3 or 6 h after treatment with ionomycin plus Q-VD.OPh, compared with the loading control, calregulin (CALR). Expression data for all samples at all time points post exposure to ionomycin in hours (h) are expressed relative to EBV-positive BL clones at time 0. Statistical significance was determined using a two-tailed Student’s *T*-test, **P<0.01, *P<0.05 and ns is not significant

Few changes were observed in response to ionomycin at early time points. However, by 48 h, 15/93 transcripts were found to be significantly differentially expressed in treated EBV-loss clones compared with their untreated counterparts, yet only 2/93 had changed significantly in EBV-positive clones compared with their untreated counterparts (Figures 6a and b and Supplementary Table 1). Although CASP8AP2 and DEDD2 were found to differ significantly in expression between all EBV-loss and EBV-positive BL clones following ionomycin treatment (Figure 6b), these changes did not validate at the protein level (Supplementary Figure 14). Comparing differentially expressed transcripts across all groups and time points demonstrated that the response of all BL clones to ionomycin was qualitatively similar, but delayed in EBV-positive clones compared with EBV-loss clones (Figure 6c).

These experiments indicate that the effects of EBV in BL are not exclusively at the transcriptional level. Therefore, the expression of 17 known apoptosis-associated proteins was quantitated by immunoblotting in EBV-positive and EBV-loss Kem-BL clones following treatment with ionomycin for 48 h in the presence of Q-VD.OPh. Following exposure to death stimuli, most proteins were similarly expressed in all clones, irrespective of EBV status (Figure 6d). Strikingly though, the proapoptotic BH3-only proteins, BIM and PUMA, were more highly induced in EBV-loss clones than their EBV-positive BL counterparts (Figure 6e). This difference was also evident at early time points, detected by FACS and immunoblotting, but only apparent following exposure to an apoptotic stimulus, not under steady-state conditions (Figure 6f).

### Functional analyses of the BCL-2 protein family in EBV-positive *versus* EBV-loss clones in three endemic BL backgrounds

**Figure 7:**
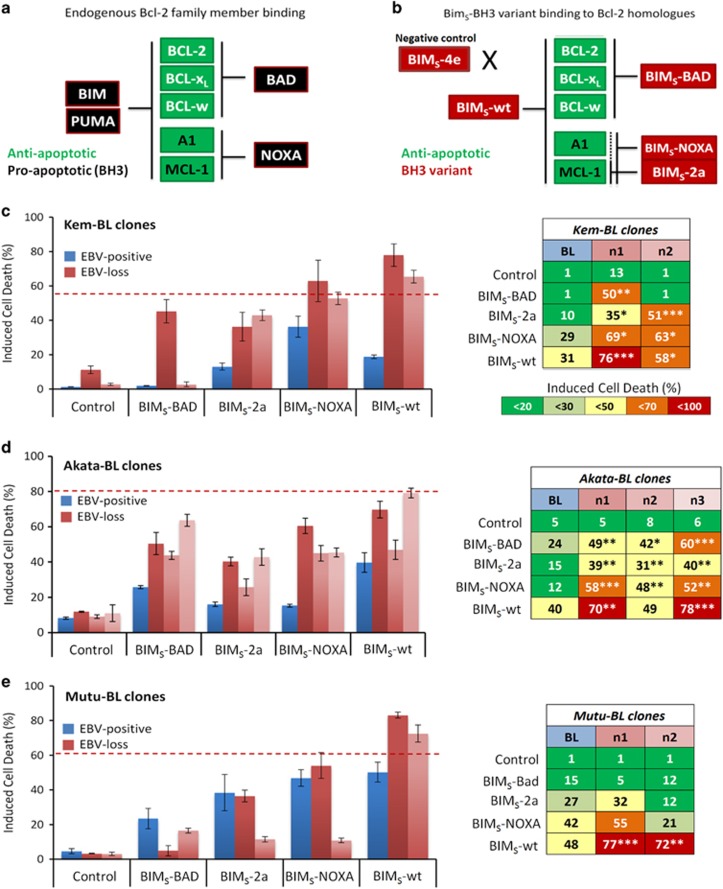
Apoptotic phenotype of EBV-positive and EBV-loss clones of BL clones expressing dox-inducible BIM_S_-BH3 variants. (**a**) Binding specificity of endogenous prosurvival BCL-2 family members (green) to proapoptotic BH3-only proteins (black). (**b**) Binding specificity of BIM_S_-BH3 variants (red) to the different prosurvival BCL-2 family members (green), dotted line indicates weak binding of BIM_S_-2a to A1/BFL1. (**c–e**) Apoptosis in EBV-positive (blue) *versus* EBV-loss clones (red) of Kem-BL (**c**), Akata-BL (**d**) and Mutu-BL (**e**) expressing BIM_S_ variants; BIM_S_-4e (negative control), BIM_S_-BAD, BIM_S_-2a, BIM_S_-NOXA and BIM_S_-wt. The left panel shows representative data (mean and S.D.) for a single experiment and the right-hand panel shows mean values from three independent experiments. Induced cell death was calculated relative to cell death in untreated control cells. All samples were treated with doxycycline to activate BIM_S_ variant expression and induce cell death. Viable cells were defined as Annexin-V/propidium iodide (PI) double negative. Statistical significance was determined using a two-tailed Student’s *T*-test to compare cell survival in each EBV-loss clone to the EBV-positive control in response to each BIM_S_-BH3 variant. Where a variant induced significantly more death in an EBV-loss clone compared with the EBV-positive control, this is noted in the right-hand panel, ***P<0.001, **P<0.01 and *P<0.05. The average amount of death induced by ionomycin treatment in EBV-loss clones from each tumour background is denoted by a dashed red line for comparison

To test if the upregulation of BIM and PUMA following apoptotic stimulation was functionally relevant across all BL backgrounds, a panel of variants based on the BIM_S_ sequence designed to recapitulate the binding and neutralisation properties of various BH3-only proteins to their BCL-2 prosurvival proteins was utilised.^47, 48^ In this system, the BIM_S_-4e protein is an inactive variant, unable to bind any prosurvival BCL-2-like protein. The BIM_S_-wt protein can bind and inhibit all BCL-2 prosurvival proteins, and therefore recapitulates the action of BIM and PUMA, which are both similarly potent inducers of apoptosis. The BIM_S_-BAD variant can bind to BCL-2, BCL-XL and BCL-W; the BIM_S_-NOXA variant can bind to MCL-1 and A1; and the BIM_S_-2a variant is a more specific inhibitor of MCL-1 (Figures 7a and b and Supplementary Table 2). The BIM_S_ variants were expressed in ten EBV-positive and EBV-loss clones across three BL backgrounds.

These assays corroborated the earlier results that implicated EBV in protecting against intrinsic apoptosis. EBV-loss clones were generally more sensitive to all BIM_S_-BH3 variants than their EBV-positive counterparts, suggesting that EBV is not acting on any individual BCL-2-like prosurvival protein. However, BIM_S_-wt, a functional mimetic of BIM and PUMA, consistently and significantly sensitised EBV-loss clones to cell death, compared with the EBV-positive BL controls (Figures 7c–e). The Akata-BL clone n2 (which remained apoptosis sensitive even after EBV reinfection) was an exception, suggesting that this clone has acquired additional genetic changes. For further verification, the phenotypic consequence of shRNA depletion of BIM and PUMA in EBV-loss and EBV-positive clones of Kem-BL and Akata-BL was analysed. Only a modest reduction in BIM and PUMA protein was achieved, yet this conferred protection from ionomycin-induced apoptosis specifically to EBV-loss clones. In EBV-positive BL clones, shRNAs targeting BIM and PUMA had no effect on cell survival, likely because BIM and PUMA are already repressed by the Latency I genes (Supplementary Figures 15 and 16).

Overall, the data provide functional confirmation that the loss of EBV-mediated inhibition of BIM and PUMA expression and proapoptotic function is responsible for the increased apoptosis sensitivity of EBV-loss BL clones. This indicates that EBV Latency I genes act in a cooperative manner to inhibit apoptosis by repressing the proapoptotic BH3-only proteins BIM and PUMA.

## Discussion

To our knowledge, this is the most extensive analysis of the role of EBV in BL. The large-scale, single-cell cloning of BL cell lines and isolation of only 61 spontaneous EBV-loss clones from >1800 clones strongly indicate that EBV is highly selected for in BL. Consistent with reports that EBV’s role in BL is to protect cells from death stimuli,^10, 17, 18, 22, 28^ our study showed that compared to isogenic EBV-positive counterparts, EBV-loss clones from multiple tumour backgrounds are consistently more sensitive to death stimuli that act through the intrinsic apoptotic pathway. Additionally, EBV loss significantly reduces the tumorigenic potential of BL cells in a xenograft mouse model of BL. Collectively, these findings demonstrate that EBV makes an ongoing contribution to the aggressive neoplastic phenotype of BL and challenges the idea that EBV only contributes to the early stages of lymphomagenesis by a ‘hit-and-run’ mechanism.^49, 50^ This work also supports the idea that enforced loss of viral genomes, perhaps in the future through drugs or EBV genome-specific CRISPR (recently demonstrated in BL^51^), could be useful clinically in the treatment of EBV-positive BLs.

Importantly, reinfection of EBV-loss clones with recombinant Ak-V could fully restore apoptosis resistance, confirming that EBV is directly responsible for the increased resistance to cell death. Interestingly, B95.8-derived rEBV, which harbours a large genomic deletion, could partially, but never fully restore apoptosis protection to BL cells. This genomic deletion spans not only a number of the miR-BARTs^42, 43^ but also a number of transcripts of unknown function that appear to be highly transcribed in latent BL cells.^52, 53^ Therefore, it is likely that genes encoded within the B95.8 deletion contribute to Latency I-mediated protection from apoptosis. Further development of rEBV genomes is required to decode the precise contribution of these complex transcripts to BL.

No Latency I-encoded gene product alone (EBNA1, EBERs or miR-BARTs) could restore apoptosis resistance to EBV-loss BLs. This indicates a requirement for cooperation between multiple genes, including transcripts from the B95.8 deletion. There is much debate in the literature in this regard. EBNA1 is essential for virus genome maintenance and also reported to function in cell survival in certain circumstances.^31^ EBER RNAs were shown to confer tumorigenicity to EBV-loss Akata-BL *in vivo* and to enhance the growth properties of cells *in vitro*, in part through upregulation of BCL-2 and IL-10.^28, 54^ Yet we found no evidence that BCL-2 or IL-10 were more highly expressed in EBER-expressing or EBV-positive cells compared with EBV-loss cells. Consistent with our findings, others reported that expression of EBERs alone did not restore protection from apoptosis in Akata-BL cells *in vitro*, but that coexpression of EBNA1 and EBERs rendered two EBV-loss Akata-BL clones more tumorigenic *in vivo*.^25^ The authors attributed this to increased EBER expression in the EBNA1-/EBER-positive EBV-loss cells. However, an alternative interpretation is that EBNA1 enhances the tumorigenicity of EBER-expressing cells by functional cooperation. Another report attributed apoptosis protection to the miR-BARTs,^18^ but the considerable clonal variation (between cell lines and viruses) in this study precludes firm conclusions. BART miRs have been reported to downregulate PUMA in epithelial cells^36^ (a finding we could recapitulate in 293 cells), but consistent with other groups,^18, 55, 56^ this downregulation was not apparent in B-lineage-derived cell lines.

Our work implicates the BH3-only proteins BIM and PUMA as the key cellular genes regulated by EBV in Latency I BL. These proteins are critical initiators of the intrinsic apoptotic pathway, able to bind and inhibit all BCL-2 prosurvival proteins and also capable of directly activating the executioners of apoptosis, BAX and BAK.^3^ In addition to the upregulation of BIM and PUMA proteins after apoptosis induction in EBV-loss cells, increased expression of BIM and PUMA transcripts was also observed compared with EBV-positive cells, although this did not reach statistical significance. Consistent with cooperation between multiple EBV genes, we therefore hypothesise that the repression of BIM and PUMA by EBV occurs at both the protein and RNA levels and involves multiple molecular mechanisms.

BART miRs are predicted to bind multiple sites in the 3′-UTRs of BIM and PUMA^36, 37, 55, 56^ and therefore likely inhibit their translation. Additionally, EBERs have been shown to activate AKT/PI3K signalling,^57^ which is important for BL cell survival.^58^ Interestingly, tonic AKT/PI3K signalling has been reported to suppress induction of PUMA and BIM, by both transcriptional and post-translational mechanisms, and consequently suppress apoptosis in leukaemia cells following growth factor withdrawal.^59^ Furthermore, EBNA1 is able to deplete cells of SMAD2,^60, 61^ which can lead to inhibition of PUMA expression through the TGF-*β* pathway.^62, 63^ A recent study showed that both RNA and DNA viruses, typified by Semliki Forest virus and herpes simplex 1 virus, respectively, control intrinsic apoptosis via regulation of PUMA.^64^ Therefore, it is likely that Latency I EBV genes co-operate to regulate PUMA and BIM both directly and indirectly and that multilateral regulation of prodeath BH3s is critical and conserved in human viruses.

The blocking of BIM and PUMA upregulation by EBV has important implications for BL pathogenesis as it has been shown that deregulated c-MYC expression can sensitise cells to apoptosis and that this predisposition must be overcome for BL to develop.^65^ Therefore, EBV infection of a preneoplastic cell bearing a c-*MYC* chromosomal translocation would be predicted to accelerate tumour development by blocking BIM and PUMA upregulation. Our work opens up the possibility to incorporate BH3-mimetic drugs (reviewed in Cory *et al.*^66^) into the treatment regimen for EBV-positive BL to help improve patient survival, which remains woeful in certain patient groups.

## Materials and methods

### Cell lines and cell maintenance

Akata-BL was a kind gift from Prof. Kenzo Takada, Rael-BL and the lymphoblastoid cell line (LCL), Raji-BL, X50-7, were kind gifts from Prof. George Klein. All other BL cell lines were established by Prof. Alan Rickinson and co-workers. Jurkat clone E6.1 was from the European Collection of Cell Cultures, HEK-293 cells were obtained from the American Tissue Culture Collection (product number: ATCC CRL-1573) and 293FT cells (R700-07) were from Life Technologies (Carlsbad, CA, USA). Clonal cell lines were established by single-cell cloning carried out as described previously.^9^ When referring to BL-derived subclones, the prefix ‘P’ indicates an EBV-positive clone, whereas ‘n’ denotes a clone that has lost the virus. BL cell lines were maintained in RPMI-1640 supplemented with 10% FCS (Gibco, Gaithersburg, MD, USA), 6 mM glutamine, 1 mM pyruvate, 50 *μ*M *α*-thioglycerol, 20 nM bathocupronine disulphonic acid and 8 *μ*g/ml gentamycin (all Sigma, St Louis, MO, USA). All cells were routinely grown at 37 °C in a humidified atmosphere containing 5% CO_2_.

### Apoptosis assays

BL cells were seeded at 9x10^4^/cm^2^ in the presence of inducers or inhibitors of apoptosis, including: ionomycin (1 *μ*g/ml, 48 h); etoposide (50 *μ*M, 24 h); Fas-activating CH11 antibodies (50 ng/ml, 24 h) (all Sigma); roscovitine (50 *μ*M, 48 h); staurosporine (250 nM, 24 h) (both Cell Signalling Technologies (CST), Danvers, MA, USA); IgM crosslinking antibodies (5 *μ*g/ml) (MP Biomedicals, Santa Ana, CA, USA); the human cytokine, IL-10 (50–500 ng/ml) (Peprotech, Rocky Hill, NJ, USA); Q-VD.OPh (20 *μ*M) (MP Biomedicals) and/or a vehicle-only control (DMSO) as stated, unless otherwise specified in figure legends. Where inducible lentiviruses were used, gene of interest expression was induced by the addition of 1 *μ*g/ml doxycycline 24 h before the experiment set-up. All assays were carried out in triplicate and on at least three occasions. Data are expressed as mean and standard deviation of independent triplicates unless otherwise specified. Cell viability was determined by dual staining of cells with Annexin-V-APC and PI or PI staining alone and analysed by flow cytometry. Surface CD95 staining was determined using the DX2-PE-CY7 (BioLegend, San Diego, CA, USA) as per the manufacturer’s instructions. A minimum of 10 000 events was recorded for all samples and data were analysed using the FlowJo Software (Treestar, OR, USA) or Accuri C6 Software (Becton Dickinson, Franklin Lakes, NJ, USA).

### Immunoblotting

Proteins in whole-cell lysates were separated by SDS-PAGE and transferred to PVDF membranes that were probed using antibodies raised against: EBNA1 (AMo serum); EBNA2 (PE2); LMP1 (CS1-4); BHRF1 (5B11); *β*-actin (clone AC-15; Sigma); caspase-3, -7 and -9 (nos. 9662, 9492, 9502; all CST); DEDD2/FLAME3 (14574; Proteintech, Chicago, IL, USA); PUMA (D30C10; CST); BIM (no. 2819; CST); CFLAR (10394-1-AP; Proteintech); CASP8AP2 (PA5-19954; Life Technologies); BID (no. 2002; CST); BAD (no. 9292; CST); BCL-2 (C-2; Santa Cruz Biotechnology (SCBT, Dallas, TX, USA)); BCL-X (H-5; SCBT); MCL-1 (no. 4572; CST); cIAP1 (no. 4952; CST), Livin (D61D1; CST); Survivin (71G4B7; CST); XIAP (3B6; CST); NOXA (114C307; Abcam, Cambridge, UK); BAK (N-20; SCBT); and BAX (N-20; SCBT). Protein size analysis and densitometry were carried out using the Chemidoc MP system equipped with the ImageLab v.5.2 Software (Bio-Rad, Hercules, CA, USA). Size markers were SeeBlue Plus 2 (Life Technologies) and densitometry calculations were normalised to endogenous control proteins, calregulin or *β*-actin.

### Animal work

Mice were kept in specified pathogen-fee animal areas at the Walter and Eliza Hall Institute of Medical Research (WEHI). All experiments involving mice were conducted in accordance with the requirements of the WEHI Animal Ethics Committee. Eight-week-old, female NSG (NOD.*Cg-Prkdc*^*scid*^*il2rg*^*tm1Wjl*^/SzJ) mice were given intraperitoneal or subcutaneous injections of BL cells diluted in PBS. Cell numbers used were 1 × 10^6^ for Kem-BL, Awia-BL and Mutu-BL clones or 1x10^7^ for Akata-BL. Mice were monitored for signs of tumour growth and killed when their tumour burden reached 1 cm^3^. Lymphoma cell suspensions made from explanted tumour tissues were shown to be >99% positive for human CD19 FACS (555415; BD Pharmingen, San Jose, CA, USA) to confirm that tumours were derived from xenotransplanted BL cells.

### Northern blotting

RNA was isolated using Trizol reagent, separated on precast urea gels (both Life Technologies) and blotted onto nylon membranes. Radio-labelled DNA probes were generated by PCR amplification of the *Eco*RI J fragment of the EBV genome using forward primer 5′-TGCTAGGGAGGAGACGTGTGT-3′ and reverse primer 5′-GAATCCTGACTTGCAAATGCTCTA-3′, followed by column purification and nick translation (Roche, Basel, Switzerland) in the presence of ^32^P-labelled dCTP (Hartmann Analytic, Braunschweig, Germany). Probe hybridisation and washes were carried out using the XpressHyb System (Clontech, CA, USA) according to the manufacturer’s instructions. Exposures were carried out at −80 °C for 2–14 days.

### Quantitative PCR analysis

Total RNA was prepared using miRVana RNA Extraction Kit (Life Technologies) according to the manufacturer’s instructions. Cellular apoptosis-related gene expression was quantified using TaqMan Low Density Array human apoptosis panel cards (4378701; Life Technologies). Random hexamer-primed cDNA was prepared using the qScript cDNA Supermix (Quanta Biosciences, Beverly, MA, USA). All experiments were carried out in triplicate and samples were run in triplicate. Data were analysed using Data Assist v.3.01 (Life Technologies) using the ΔΔCt method, and then normalised to the mean of two endogenous controls, *ACTB* and *GAPDH*. Absolute quantitation of EBV transcripts and housekeeping controls were quantitated by real-time PCR relative to a DNA plasmid standard to enable absolute copy numbers to be calculated. This method and all the relevant primer-probe sets have been described elsewhere.^53^ Stem-loop primed cDNA was prepared with miR-specific primers and TaqMan microRNA Reverse Transcription Kit (Life Technologies) according to the manufacturer’s guidelines and miR expression was quantified using commercially available TaqMan primer/probe mixes (Life Technologies). EBV genome load was calculated by DNA q-PCR. Briefly, DNA was extracted using the GenElute DNA Kit (Sigma) according to the manufacturer’s instructions and 5 ng DNA was used per q-PCR assay. Genome load was calculated by quantitating copies of the EBV polymerase gene (*BALF5*) normalised to *β*-2-microglobulin (*β2M*), assuming two genomic copies of *β2M* per cell. Each measurement was made in triplicate.

### Plasmids, lentiviruses, recombinant EBVs and generation of stable cell lines

The EBNA1 protein, clusters of BART miRs and BIM_S_-variant proteins were expressed from an F-UTG-derived lentivirus, which constitutively expresses GFP and drives expression of the gene of interest from a dox-inducible, polII, Trex promoter, as described previously.^67^ Recombinant Akata virus (Ak-V)-producing cells were a kind gift from Prof. Kenzo Takada and the CpWp-KO B95.8 virus (B-V) was developed by Dr. Rosemary Tierney. Detailed descriptions of all recombinant EBVs and expression constructs are available in Supplementary Information. All constructs were sequence verified.

Lentivirus containing supernatants were produced by transfecting 293FT cells (Life Technologies) with the expression constructs of interest alongside the envelope plasmid, pMD2.G, and the packaging plasmid, psPax2, using Lipofectamine 2000 (Life Technologies). Infectious virus stocks produced from B-V BAC-carrying 293 clones by BZLF1/BALF4 transfection^68^ or from Ak-V-infected BL cells by crosslinking surface IgG^40^ as described previously. Before EBV infection, EBV-loss BL clones were transduced with an expression construct expressing the EBV receptor, human CD21, to enhance the infection efficiency.^69^

### Statistical analysis

For BIM_s_-BH3 variant functional assays, reinfection studies, q-PCR and immunoblot gene expression analyses the significance of differences between cell lines were calculated using an unpaired, two-tailed, Student’s *T*-test. For apoptosis assays to characterise the effect of EBV loss, unpaired, two-tailed Student’s *T*-tests were carried out on clones from each background. Additionally, the difference in cell survival between all EBV-positive clones and all EBV-loss clones across different BL backgrounds was analysed by two-way ANOVA. Survival comparisons of mouse cohorts were calculated using log-rank (Mantel–Cox) analysis. *T*-tests, Mantel–Cox and ANOVA analyses were carried out in the GraphPad Prism 5.0 Software (La Jolla, CA, USA). Differences were considered significant where the *P*-value fell below 0.05 and were classified as follows: *P<0.05, **P<0.01, ***P<0.001 and ^ns^*P*>0.05 (not significant).

## Figures and Tables

**Figure 1:**
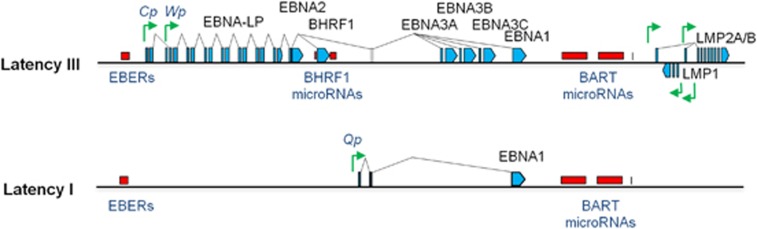
Examples of patterns of EBV gene expression. Schematic showing the Latency III EBV gene expression programme (as found in B cells transformed *in vitro* into lymphoblastoid cell lines (LCLs)) and the Latency I EBV gene expression programme (as found in the majority of EBV-positive BL tumours and cell lines derived from these tumours). Latent proteins (EBNA1, EBNA2, EBNA3A, EBNA3B, EBNA3C, EBNA-LP, BHRF1, LMP1 and LMP2A/B) are shown in blue. Non-coding RNAs (EBERs, BHRF1 microRNAs and miR-BARTs) are shown in red, and latent promoters (Cp, Wp, Qp and LMP promoters) are shown in green

**Table 1:** Summary of single-cell cloning of BL cell lines

**Cell line**	**Genome load**	**EBV-loss frequency**	
	**Range (median)**	**Number**	**Percentage**
Eli-BL	0–68 (13)	38/103	36.90
Akata-BL	0–51 (13)	15/272	5.50
Kem-BL	0–300+ (35)	3/185	1.60
Mutu-BL	0–300+ (61)	3/195	1.50
Awia-BL	0–35 (9)	2/190	1.10
Ava-BL	6–68 (39)	0/158	—
Chep-BL	2–80 (2)	0/149	—
Dante-BL	11–68 (19)	0/95	—
Oku-BL	5–37 (8)	0/135	—
Rael-BL	7–300+ (43)	0/91	—
Sal-BL	6–30 (15)	0/72	—
Sav-BL	30–300+ (231)	0/175	—

Abbreviations: BL, Burkitt lymphoma; EBV, Epstein–Barr virus

**Table 2:**
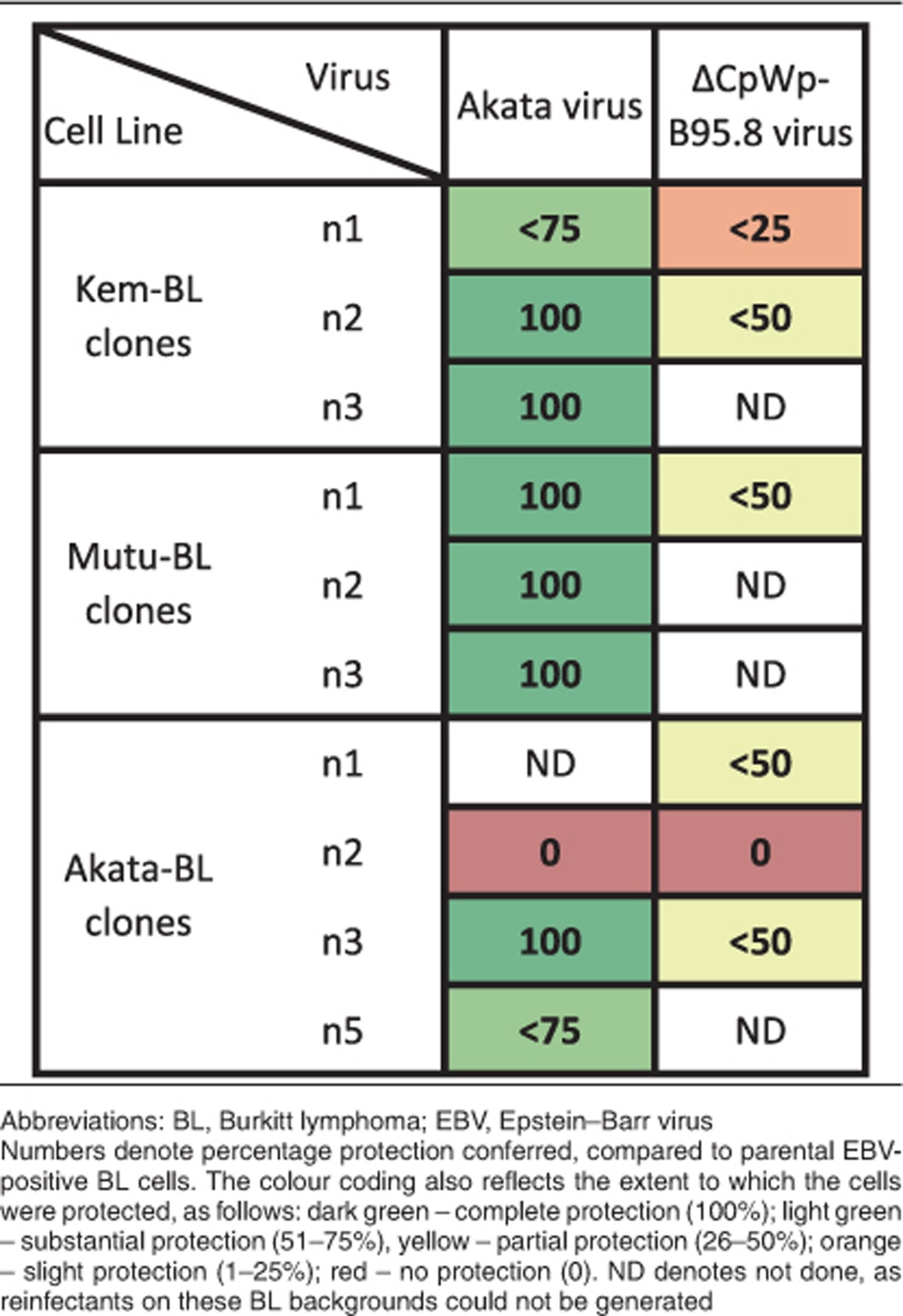
Summary of apoptosis sensitivity in EBV-loss clones derived from three BL backgrounds reinfected with ΔCpWp-B95.8 rEBV or the Akata virus strain
