# Supplementary material for: Coordinated repression of BIM and PUMA by Epstein–Barr virus latent genes maintains the survival of Burkitt lymphoma cells
Source: Cell Death Differ. 2017 Sep 29;25(2):241–54. doi: 10.1038/cdd.2017.150 (PMC5762840; doi:10.1038/cdd.2017.150)
Supplement: Supplementary Information [file cdd2017150x1.pdf]

BH3 protein repression in Burkitt lymphoma

SUPPLEMENTARY INFORMATION

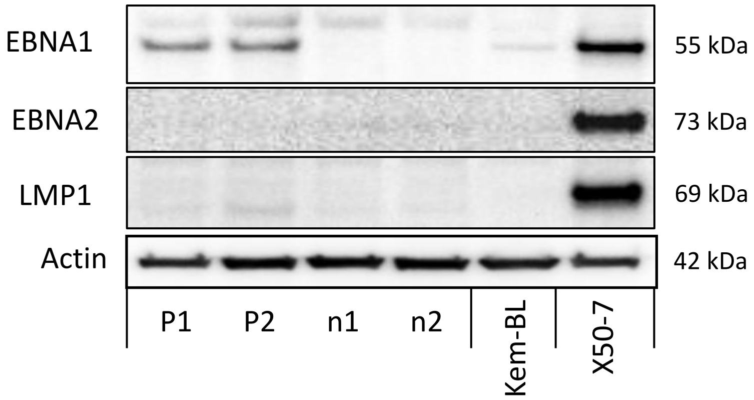

SI Figure 1. EBV latent protein expression in EBV-positive and EBV-loss Awia-BL clones

Expression of EBV latent proteins, EBNA1, EBNA2 and LMP1 in isogenic EBV-positive (P1-P2) and EBV-loss (n1-n2) Awia-BL clones compared to Latency I (Kem-BL) and Latency III (X50-7) controls. Probing for Actin was used as a loading control.

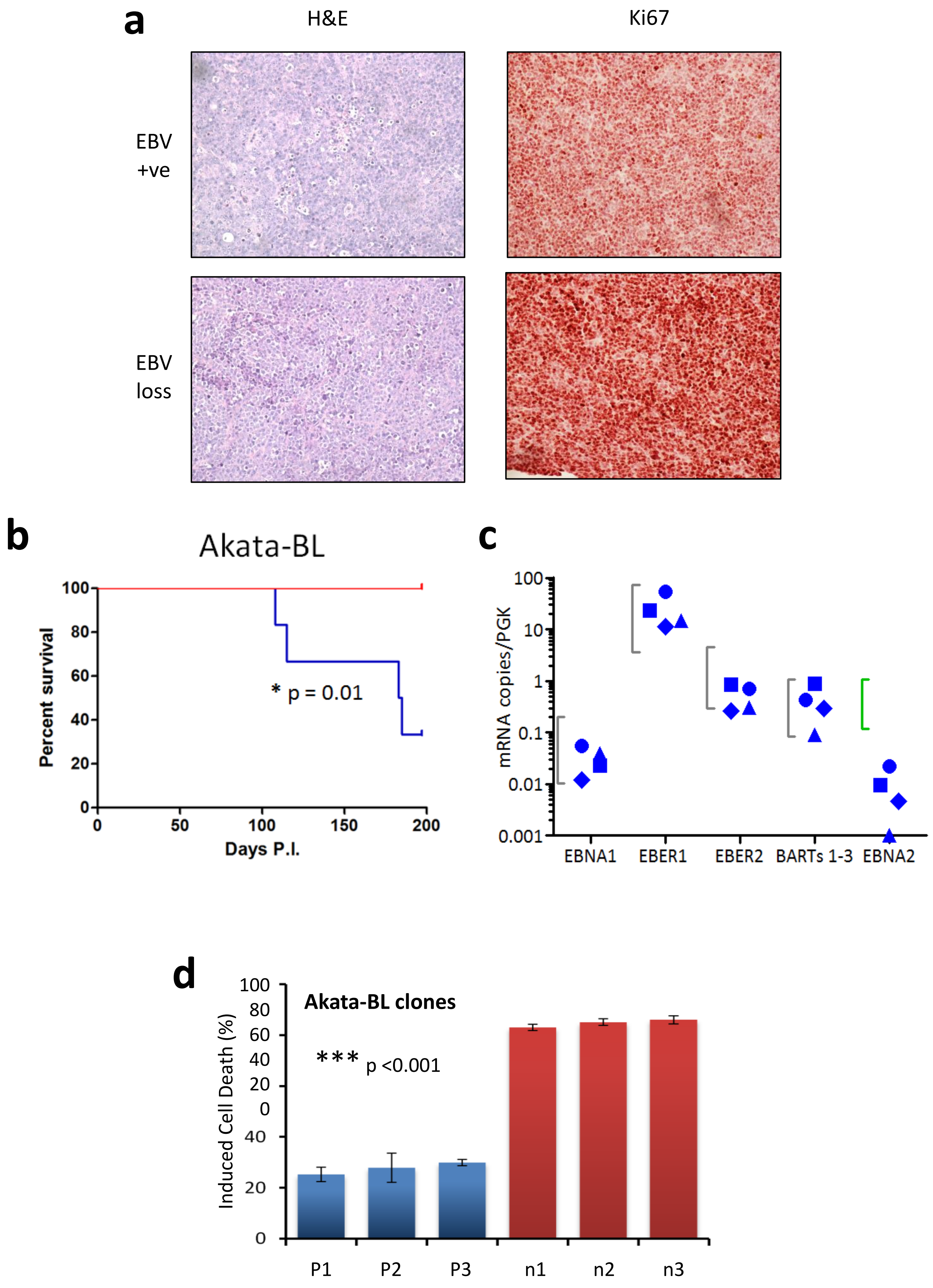

SI Figure 2. Extended data on phenotype and genotype of BLs in vivo and in vitro

(a) Sections from tumours from NSG BL xenograft mice. Examples of starry sky patterning in H&E stain (left) and staining with the proliferation maker, Ki67, (right) in ovarian tumours from mice injected with EBV-positive (upper panels) or EBV-loss (lower panels) Kem-BL cells. Images taken at 20x magnification. (**b**) Kaplan-Meier survival plot comparing survival in days post inoculation (P.I) of NSG mice injected with EBV positive (blue, n=9) or EBV-loss (red, n=7) Akata-BL cells. (**c**) Gene expression in *ex vivo* BL xenograft derived cells from four representative animals (◆ Kem P1, ● Kem P2, ▲ Mutu P1, ■ Mutu P2). Grey brackets indicate the normal range of transcripts in the Latency I BL cell lines from which the clones were derived. Green bracket indicates the normal range of EBNA2 transcripts in a panel of four, Latency III, LCLs. EBNA1 refers to Qp-initiated transcripts and BARTs refers to primary transcripts spliced between exons 1 and 3, where the majority of the miRNAs are encoded. Data are expressed relative to the levels of the endogenous control, PGK. (**d**) Ionomycin-induced cell death in isogenic EBV-positive (P1-P3) versus EBV-loss (n1-n3) clones of Akata-BL (48 h), compared to vehicle treated controls. Data are the mean and standard deviation (SD) of pooled data from three independent experiments, each carried out in triplicate. Statistical significance was calculated using an unpaired, two-tailed, Student's T-test.

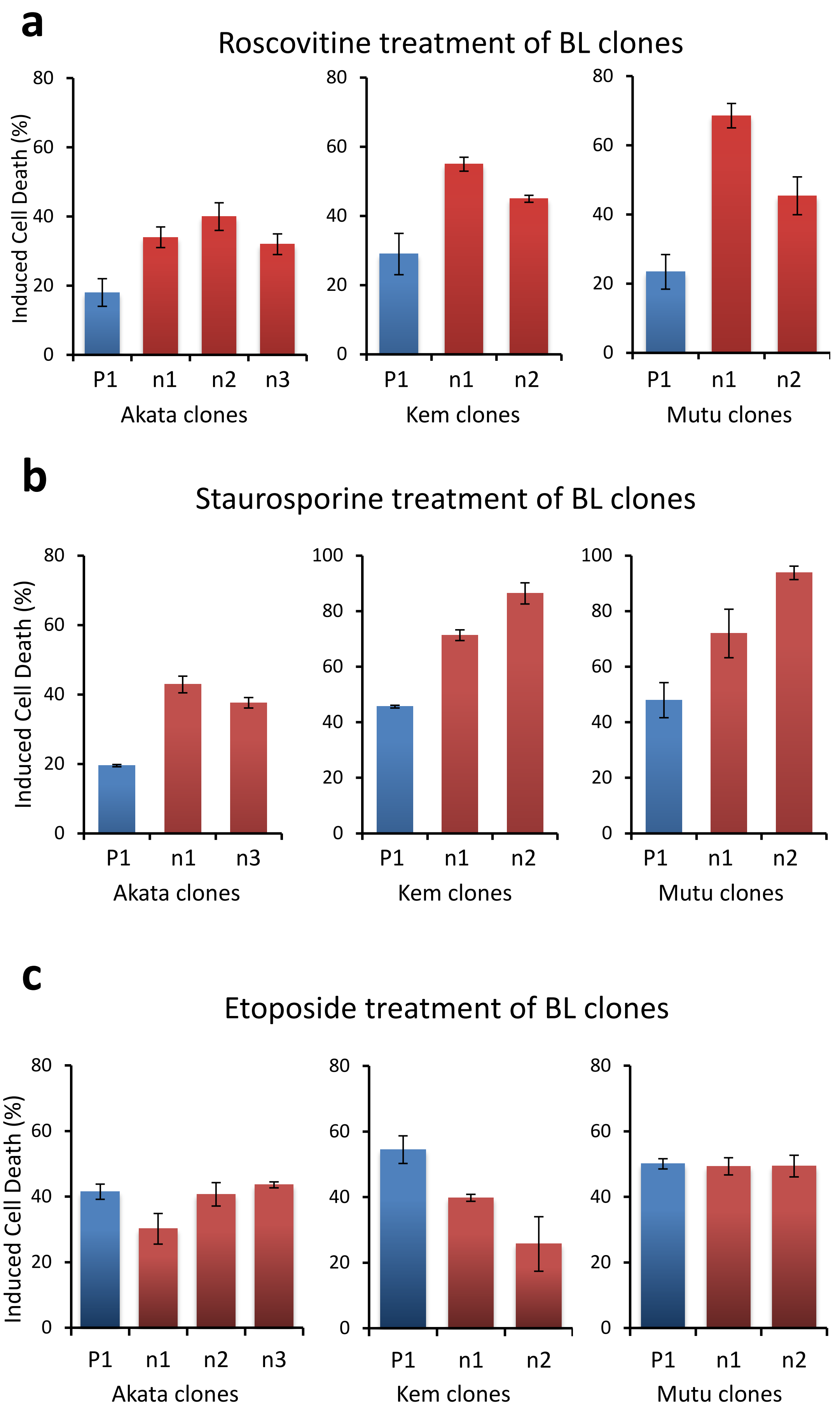

SI Figure 3. Response of EBV-positive and EBV loss BL clones to different cytotoxic agents

Cell death induced upon treatment of EBV-positive (P1) and EBV-loss (n1-n3) clones of Akata-BL, Kem-BL and Mutu-BL with cytotoxic drugs. (**a**) Clones treated with 50 µM roscovitine for 48 h. EBV-loss clones were significantly more sensitive to roscovitine than EBV-positive clones, * $p = 0.03$, in two-way ANOVA analysis. (**b**) Clones treated with 250 nM staurosporine for 24 h. EBV-loss clones were significantly more sensitive to staurosporine than EBV-positive clones, * $p = 0.02$, in two-way ANOVA analysis. (**c**) Clones treated with 50 µM etoposide for 24 h. There was no statistically significant difference between EBV-positive and loss clones in two-way ANOVA analysis. Each data point represents the mean and standard deviation (SD) from three separate experiments, each experiment was performed in triplicate. Comparisons were made relative to vehicle-only treated cells.

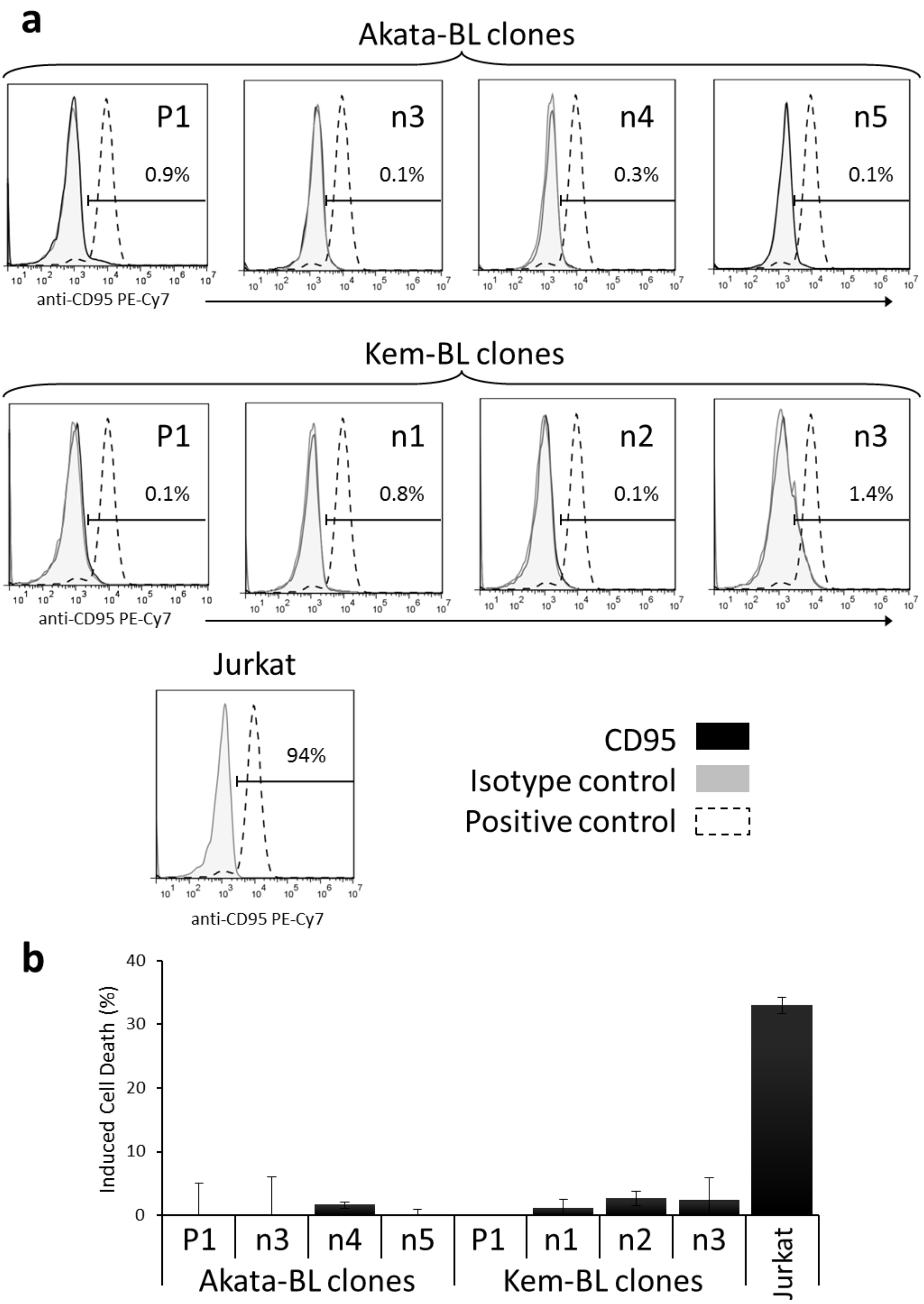

SI Figure 4. Resistance to extrinsic apoptosis in EBV-positive and loss BL clones

(a) Surface staining for Fas (CD95) in EBV-positive and loss clones of Kem-BL and Akata-BL compared to the Fas sensitive T cell lymphoma cell line, Jurkat. (**b**) Cell death induced by treatment with anti-Fas antibody (CH11) in BL clones compared to Jurkat. Data are representative of experiments carried out on three independent occasions.

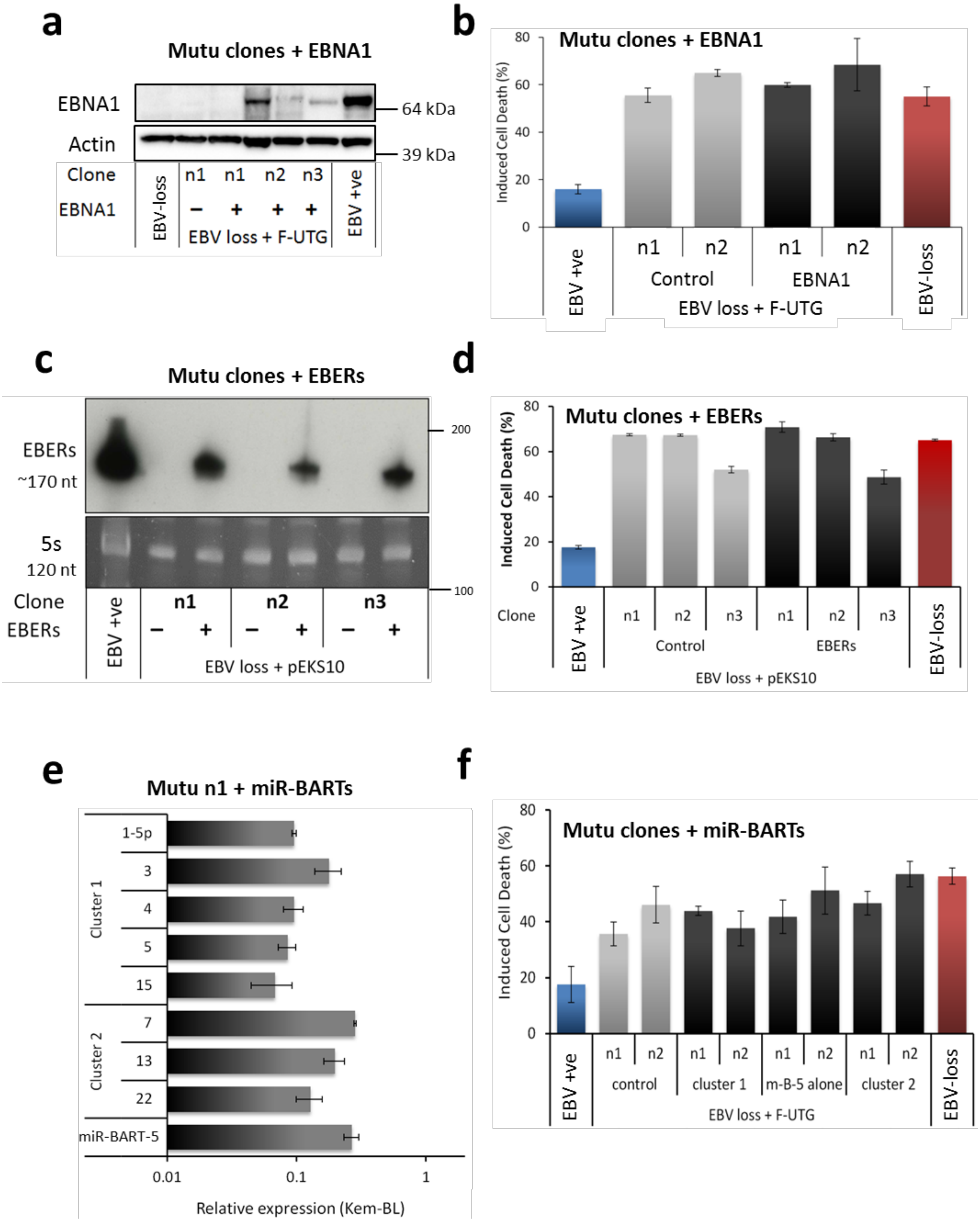

SI Figure 5. Apoptosis phenotype of EBV-loss Mutu-BL clones expressing Latency I-associated genes

EBV-loss Mutu-BL clones (n1-n3) expressing EBNA1 (**a**-**b**), EBERs (**c**-**d**) or miR-BARTs (**e**-**f**) and empty vector controls, marked (+) and (-), respectively, compared to EBV-loss (EBV-ve) and EBV-positive BL cells (EBV +ve). (**a**) EBNA1 protein expression, identified with human AMo serum using probing for actin as a loading control. (**b**) Survival of ionomycin-treated EBNA1-expressing EBV-loss BL cells compared to controls. (**c**) EBER RNA

expression identified by Northern blot analysis by probing with the *EcoRI* J fragment of the EBV genome, 5S was used as a loading control. (**d**) Survival of ionomycin-treated EBER-expressing EBV-loss BL cells compared to control cells. (**e**) Expression of mature BART miRs by q-PCR, expressed from the Cluster 1 (top), Cluster 2 (middle), or miR-BART-5 only (bottom) constructs, relative to the levels in Kem-BL cells. (**f**) Survival of ionomycin treated BART miR expressing EBV-loss BL cells compared to controls. All F-UTG transduced cells in **a**, **b**, **e** and **f** induced with 1 µg/ml dox for 24 h before experiments were carried out. In apoptosis assays (**b**, **d** and **f**), cell death was induced by treatment with 1 µg/ml ionomycin for 48 h. Data are representative of assays that were carried out in triplicate on three independent occasions.

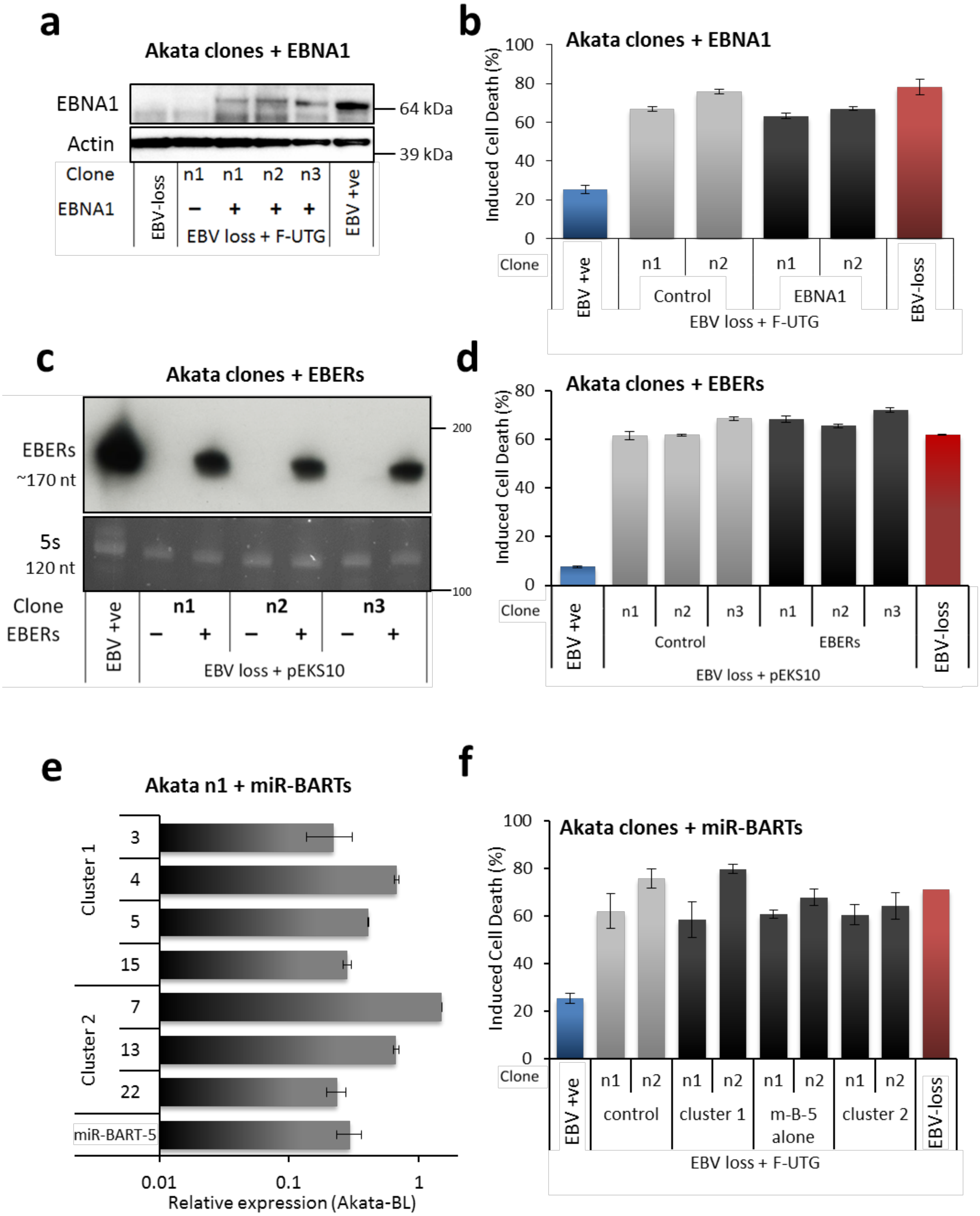

SI Figure 6. Apoptosis phenotype of EBV-loss Akata-BL clones expressing Latency I-associated genes

EBV-loss Akata-BL clones (n1-n3) expressing EBNA1 (**a**-**b**), EBERs (**c**-**d**) or *BART* miRs (**e**-**f**) and empty vector controls, marked (+) and (-), respectively, compared to EBV-loss (EBV-loss) and EBV-positive cells (EBV +ve). (**a**) Western blot to detect EBNA1 protein expression identified with human AMo serum. Probing for actin was used as a loading control. (**b**) Survival of ionomycin treated EBNA1-expressing EBV-loss BL cells

compared to controls. (**c**) EBER RNA expression by Northern blot probed with the *EcoRI* J fragment of the EBV genome. Probing for 5S was used as a loading control (**d**) Survival of ionomycin-treated EBER-expressing EBV-loss BL cells compared to controls. (**e**) q-PCR to detect expression of mature BART miRs from the Cluster 1 (top), Cluster 2 (middle), or miR-BART-5 only (bottom) constructs, relative to levels in Kem-BL (**f**) Survival of ionomycin treated BART miR-expressing EBV-loss BL cells compared to controls. All F-UTG transduced cells in **a**, **b**, **e** and **f** were induced by treatment with 1 µg/ml dox for 24 h before experiments were carried out. In apoptosis assays (**b**, **d** and **f**), cell death was induced by treatment with 1 µg/ml ionomycin for 48 h. Data are representative of assays that were carried out in triplicate on three independent occasions.

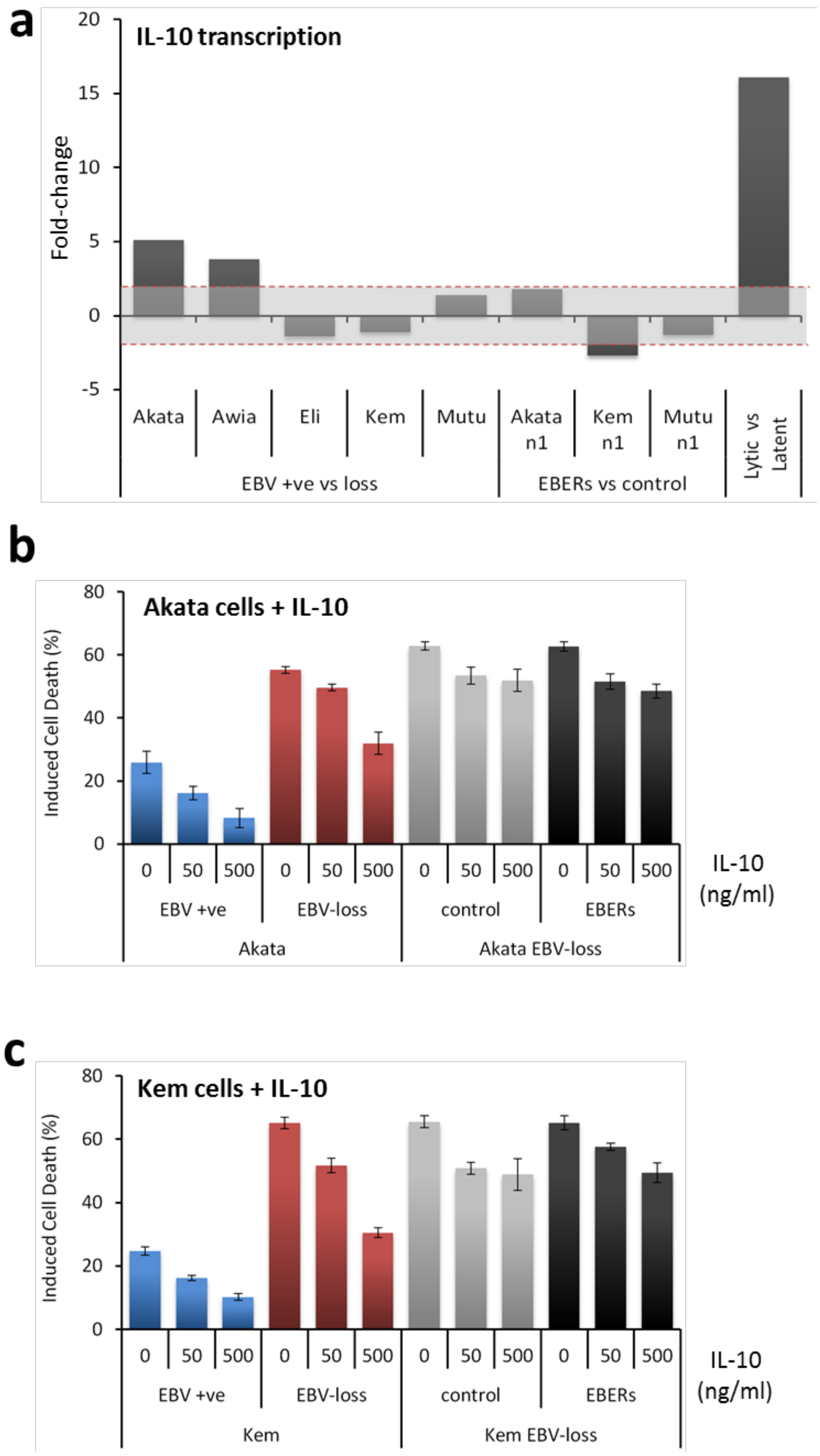

SI Figure 7. Comparison of IL-10 expression, EBER expression and apoptosis phenotype in BL cells

(a) IL-10 transcription in EBER-expressing and EBER-negative cell lines. Differences in expression, calculated as fold-change, were determined for: EBV-positive clones of Akata, Awia, Eli, Kem and Mutu BLs versus EBV-

loss clones derived from the same tumour background; EBER-expressing EBV loss clones of Akata, Kem, and Mutu BLs, versus the same EBV loss clones (Akata n1, Kem n1 and Mutu n1), containing a control vector; and 100% lytic versus tightly latent AKBM cells. All fold-changes were calculated from data from q-PCR analyses carried out in duplicate on two each of EBV positive and EBV-loss clones or EBER positive and control cell lines, respectively. AKBM cells can be efficiently induced into lytic cycle by cross-linking with IgG and express a GFP marker of lytic cycle entry that can be used to sort pure populations of lytic cells [1]. The grey shaded area denotes changes of <2-fold. (**b**) Cell death in EBV-positive Akata-BL (EBV +ve), EBV-loss clone n1 (EBV-loss), clone n1 + empty control vector (control) or clones n1 + EBER ESK10 vector (EBERs), treated for 48 h with 1 µg/ml ionomycin and IL-10 at 50 or 500 ng/ml. (**c**) Cell death in EBV-positive Kem-BL (EBV +ve), EBV-loss clone n1 (EBV-loss), clone n1 + empty control vector (control) or clones n1 + EBER ESK10 vector (EBERs), treated with 1 µg/ml ionomycin and IL-10 at 50 or 500 ng/ml for 48 h. Apoptosis data are representative of assays that were carried out in triplicate on three independent occasions.

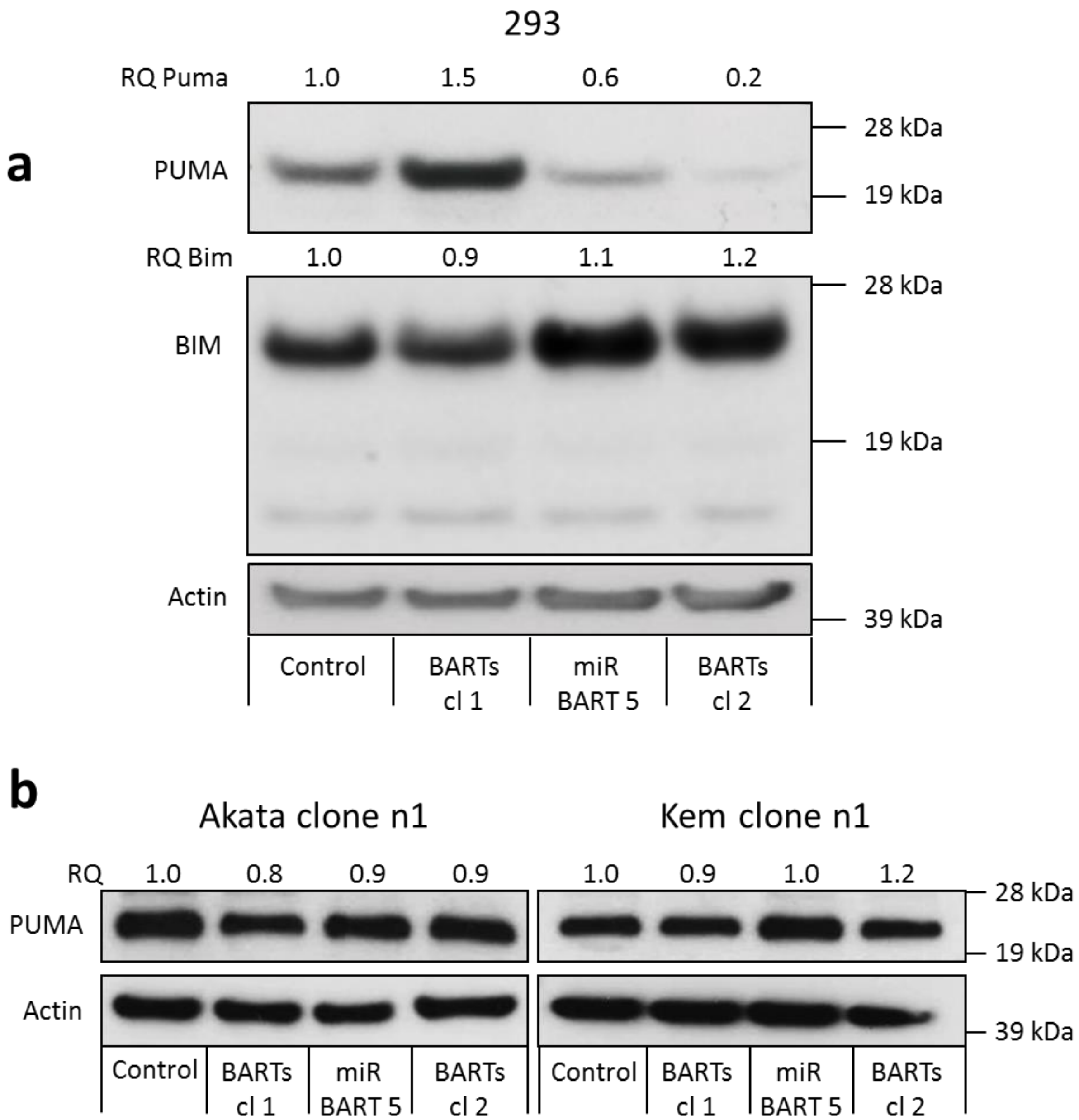

SI Figure 8. Effect of BART miRNA expression on PUMA protein in 293 cells and BL cells

(**a**) Western blots of PUMA and BIM expression in miR BART-expressing 293 cells. BART miRs were expressed as either cluster 1 (BARTs cl 1), cluster 2 (BARTs cl 2) or miR BART 5 alone (miR BART 5) using the dox-inducible F-UTG lentivirus system at comparable high levels to those detected in NPC tumours (not shown). (**b**) Western blots of PUMA expression in EBV-loss clones (Akata n1 and Kem n1) expressing miR BARTs cluster 1 (BARTs cl 1), cluster 2 (BARTs cl 2) or miR BART 5 alone (miR BART 5). Densitometry and relative quantitation (RQ) was carried out using ImageLab software (BioRad) and normalised to the loading control, β-actin (Actin). Expression of BART microRNAs was induced by addition of 1 μg/ml doxycycline to the cell growth medium 48 hours prior to analysis.

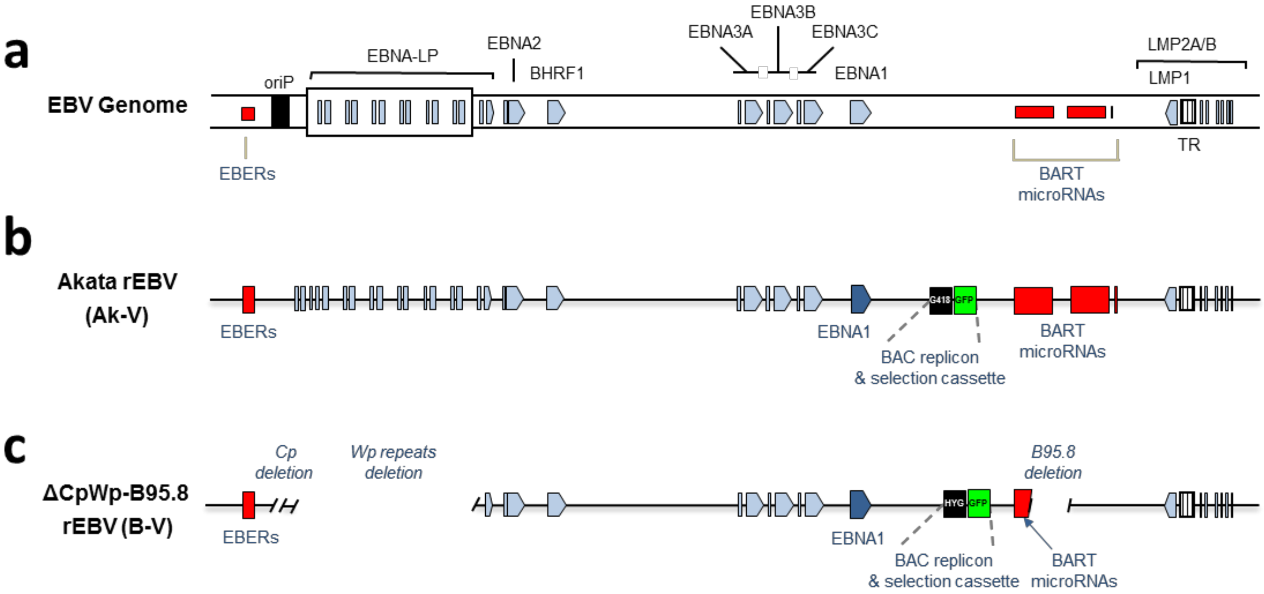

SI Figure 9. Schematic of EBV genome layout and virus strains used for reinfection experiments

(a) Layout of EBV genome, depicting latent genes (*EBNA-LP*, *EBNA1*, *EBNA2*, *EBNA3s*, *LMP1*, *LMP2A/B*, *BHRF1*, the *EBERs* and the *BART* microRNAs), the origin of lytic replication (oriP) and the terminal repeat sequences (TR). (**b**) Akata virus recombinant EBV strain showing the position of the various EBV genetic elements as well as the position of the BAC replicon, selection cassette and GFP inserted into the *BXLF1* locus, corresponding to position 131290 of the prototype EBV sequence (AJ507799) [2]. (**c**) Schematic of the ΔCpWp-B95.8 EBV BAC showing the position of EBV genetic elements, BAC replicon, selection cassette and GFP. Additionally this strain carries deletions across the Cp promoter (corresponding to positions -429 to +846 in relation to the Cp start site), all 11 copies of the tandemly repeated Wp promoter (corresponding to positions 13215-47007 of the prototype EBV sequence (AJ507799) [3, 4].

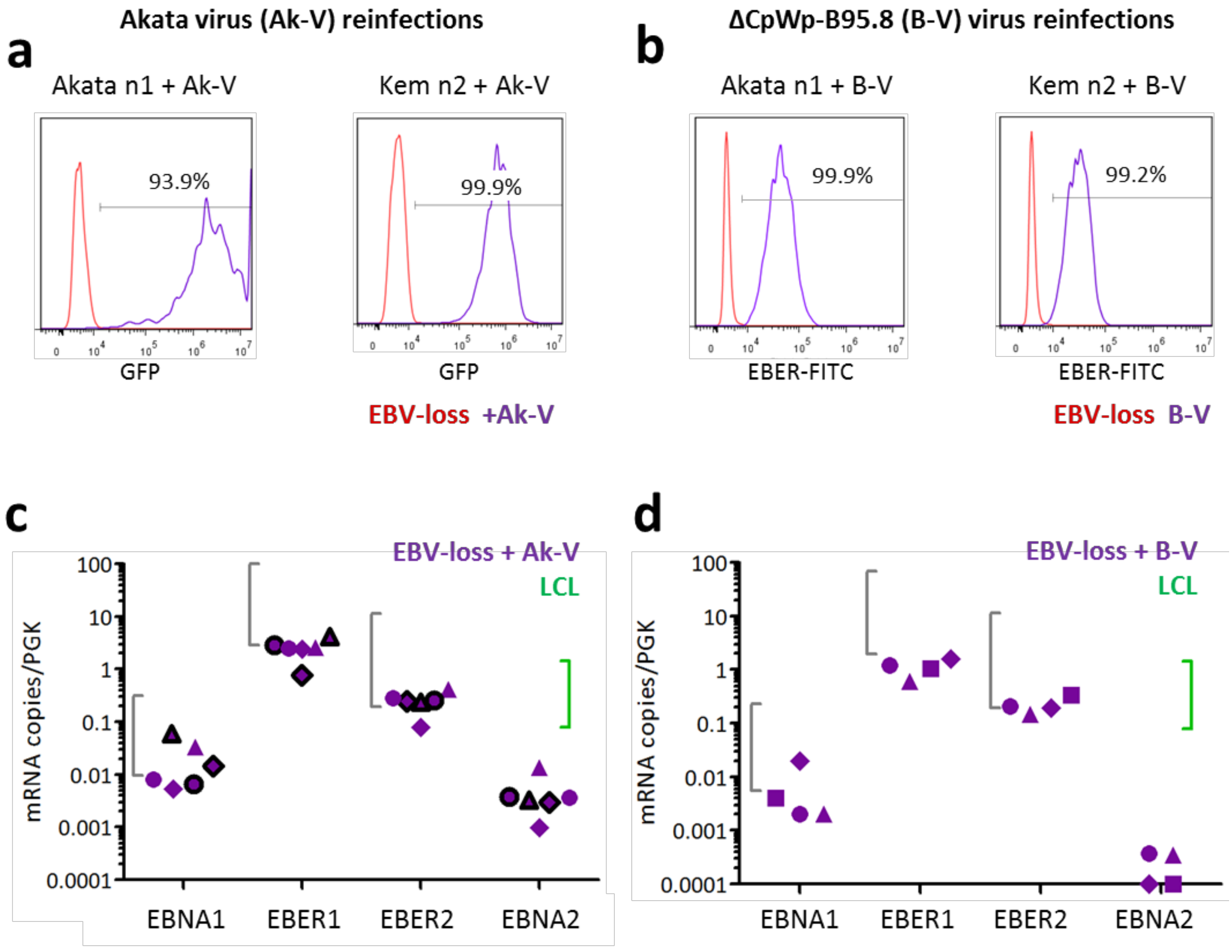

SI Figure 10. Further characterisation of reinfected EBV-loss clones

(a) GFP staining in Akata clone n1 (left) and Kem clone n2 (right) reinfected with the GFP-positive Akata virus (Ak-V) in purple, compared to uninfected cells of the same clone (red). (**b**) EBER staining by *in situ* hybridisation and analysed by flow cytometry in Akata clone n1 (left) and Kem clone n2 (right) reinfected with the ΔCpWp-B95.8 virus (B-V) in purple, compared to uninfected cells of the same clone (red). (**c**) Transcription of EBV latent RNAs in EBV-loss BL clones of re-infected with Ak-V (purple), compared to the range seen in a panel of eight Latency I BL cell lines (grey bracket), including those from which the EBV loss clones were isolated. EBNA1 refers to Q-U-K transcripts driven from the *Qp* promoter that are indicative of Latency I, EBER1 and EBER2 pick up intergenic sequences within the non-coding RNAs. EBNA2 expression is indicative of a Latency III gene expression programme, the range of EBNA2 expression in 4 Latency III LCLs (green bracket), is shown for comparison. Data are expressed relative to levels of the endogenous control, PGK. (**d**) Transcription of EBV latent RNAs in EBV-loss clones Akata n2 and Kem n1 reinfected with B-V (purple), compared to the range seen in a panel of eight Latency I BL cell lines (grey bracket) or in four Latency III LCLs (green bracket).

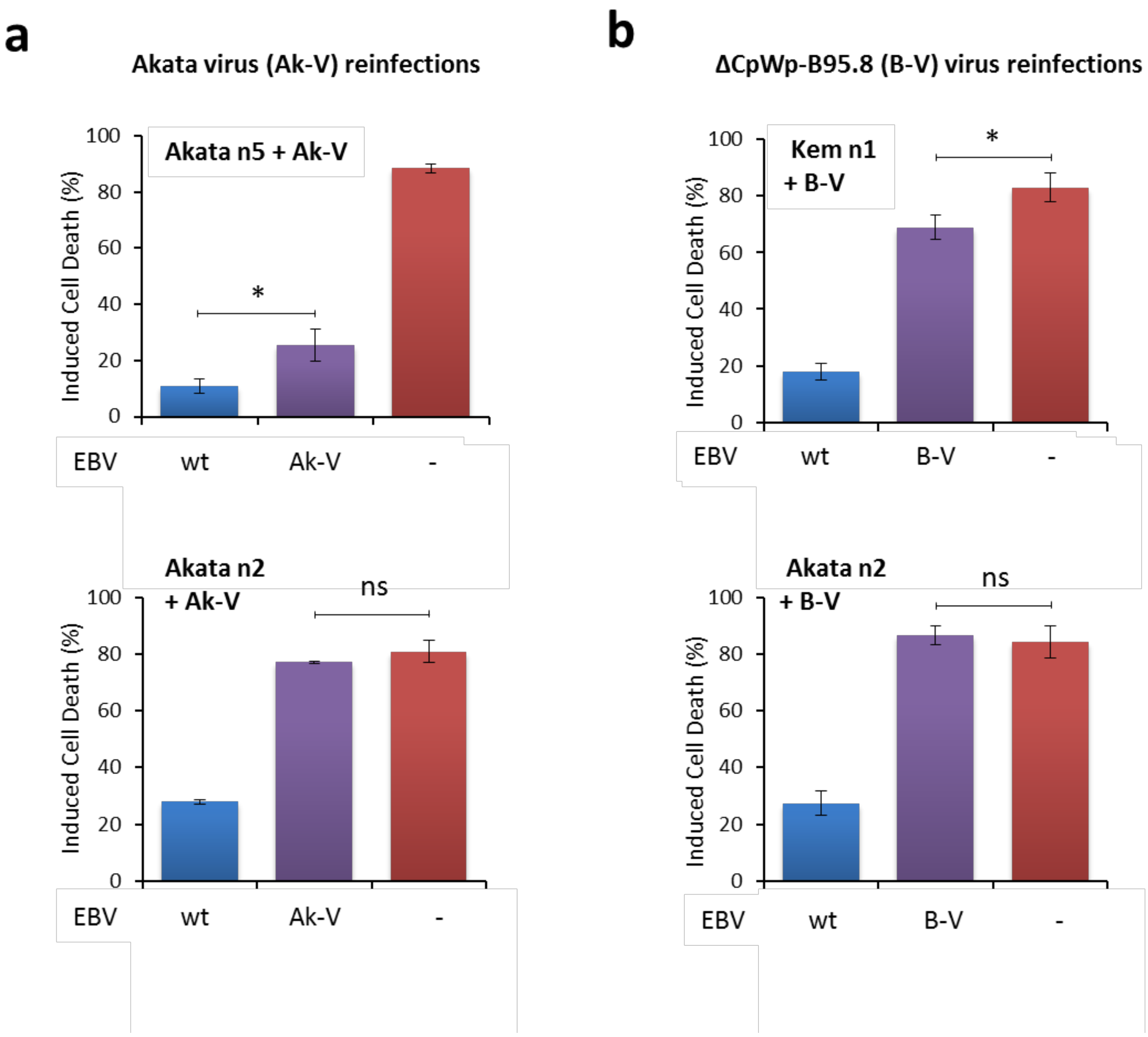

SI Figure 11. Further characterisation of the apoptosis phenotypes of reinfected EBV-loss clones

(a) Survival of EBV-loss clones re-infected with Akata virus (Ak-V, purple) after challenge with 1 µg/ml ionomycin for 48 h relative to EBV-loss cells (-, red) and compared to EBV-positive parental BL cells (wt, blue) from the same clonal background. The upper panel shows the Akata clone n5 re-infectant that was largely protected, but still significantly more sensitive than the parental cells. The lower panel shows the Akata clone n2 re-infectant that remained as sensitive as uninfected cells. (**b**) Survival of EBV-loss BL clones re-infected with ΔCpWp-B95.8 virus (B-V, purple) after challenge with 1 µg/ml ionomycin for 48 h relative to EBV-loss cells (-, red) and compared to EBV-positive parental BL cells (wt, blue) from the same clonal background . Upper panel: Kem clone n1 B-V re-infectant that was largely sensitive, but still significantly more resistant than parental cells. Lower panel: Akata clone n2 B-V re-infectant which remained as sensitive as uninfected cells. Data are presented as the mean and SD of three independent experiments, each carried out in triplicate. Statistical significance was determined using a two-tailed Student's T-test, $p < 0.05$, ns is not significant.

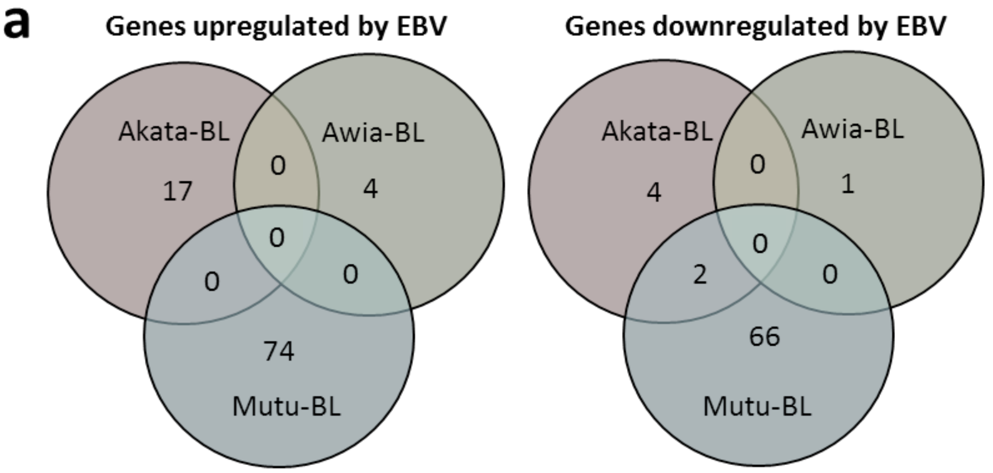

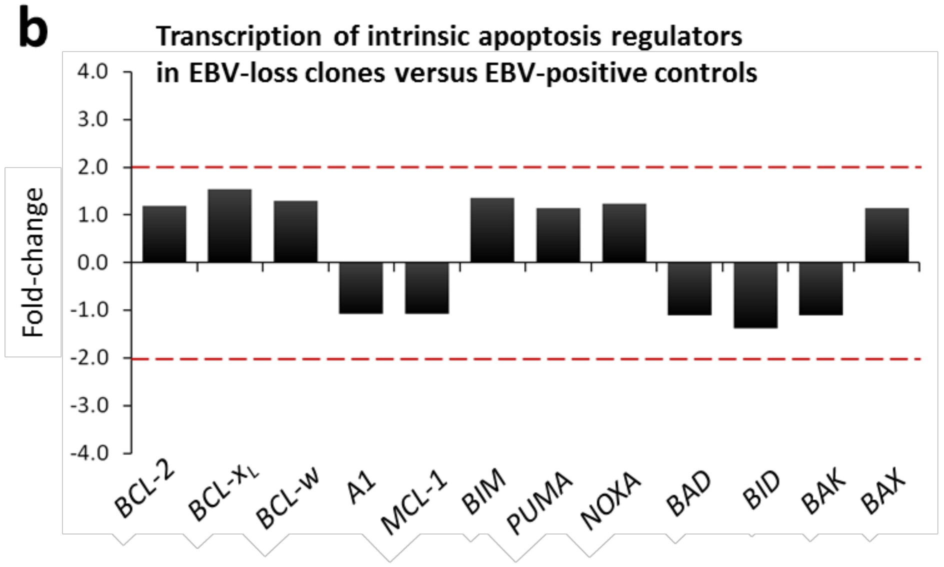

Name	Gene Symbol	Fold-change				Overall Fold Change
		Awia-BL	Akata-BL	Eli-BL	Mutu-BL	
BCL-2	*BCL2*	1.3	1.0	1.6	1.0	1.2
BCL-x_L	*BCLXL*	1.5	1.1	1.2	2.6	1.5
BCL-w	*BCL2L2*	1.6	1.0	1.4	1.2	1.3
A1	*BCL2A1*	1.2	-1.2	0.7	-1.1	-1.1
MCL-1	*MCL1*	1.1	-1.1	-1.1	-1.3	-1.1
BIM	*BCL2L11*	1.3	1.3	2.5	1.0	1.3
PUMA	*BBC3*	1.0	1.1	1.0	1.4	1.1
NOXA	*PMAIP1*	1.4	1.3	1.0	1.1	1.2
BAD	*BAD*	1.1	1.0	-1.4	-1.4	-1.1
BID	*BID*	-1.3	1.0	-2.4	-1.6	-1.4
BAK	*BAK1*	1.0	-1.1	-1.6	1.0	-1.1
BAX	*BAX*	1.2	1.2	1.1	1.0	1.1

SI Figure 12. Summary of data from microarray gene expression analysis carried out on EBV-positive and EBV-loss clones from four different BL backgrounds

(a) Complementary RNA was synthesised from EBV-positive and EBV-loss clones from the Akata-BL, Awia-BL and Mutu-BL backgrounds (two pairs of clones from each background), fragmented and hybridised to Affymetrix human genome U133 plus 2.0 arrays. Differentially expressed genes were identified using limma with tumour and EBV status as explanatory variables and with criteria of absolute fold change > 2 and false discovery rate < 10%. Clones of Kem-BL were not included in these experiments, because EBV-loss clones had not been isolated from the Kem-BL background at the time the arrays were carried out. The cells were not treated with apoptosis inducers and were >90% viable at the time of harvesting. We also compared global gene expression in all samples to the published molecular BL signature developed by Hummel and Dave and were able to confirm that all clones maintained a molcular BL index of >0.999 compared to primary, uncultured, BL tumour samples, regardless of EBV status [5, 6]. (**b**) Fold changes in transcripts of BCL2 family member mRNAs across 7 EBV-positive and 7 EBV-loss clones in Affymetrix human genome U133 plus 2.0 arrays. In addition to two EBV-positive and two EBV-loss clones of Akata-BL, Awia-BL and Mutu-BL, one EBV-positive and one EBV-loss clone derived from Eli-BL were also analysed by microarray gene expression analysis. There was no significant difference in expression of any BCL-2 family member across the four BL backgrounds.

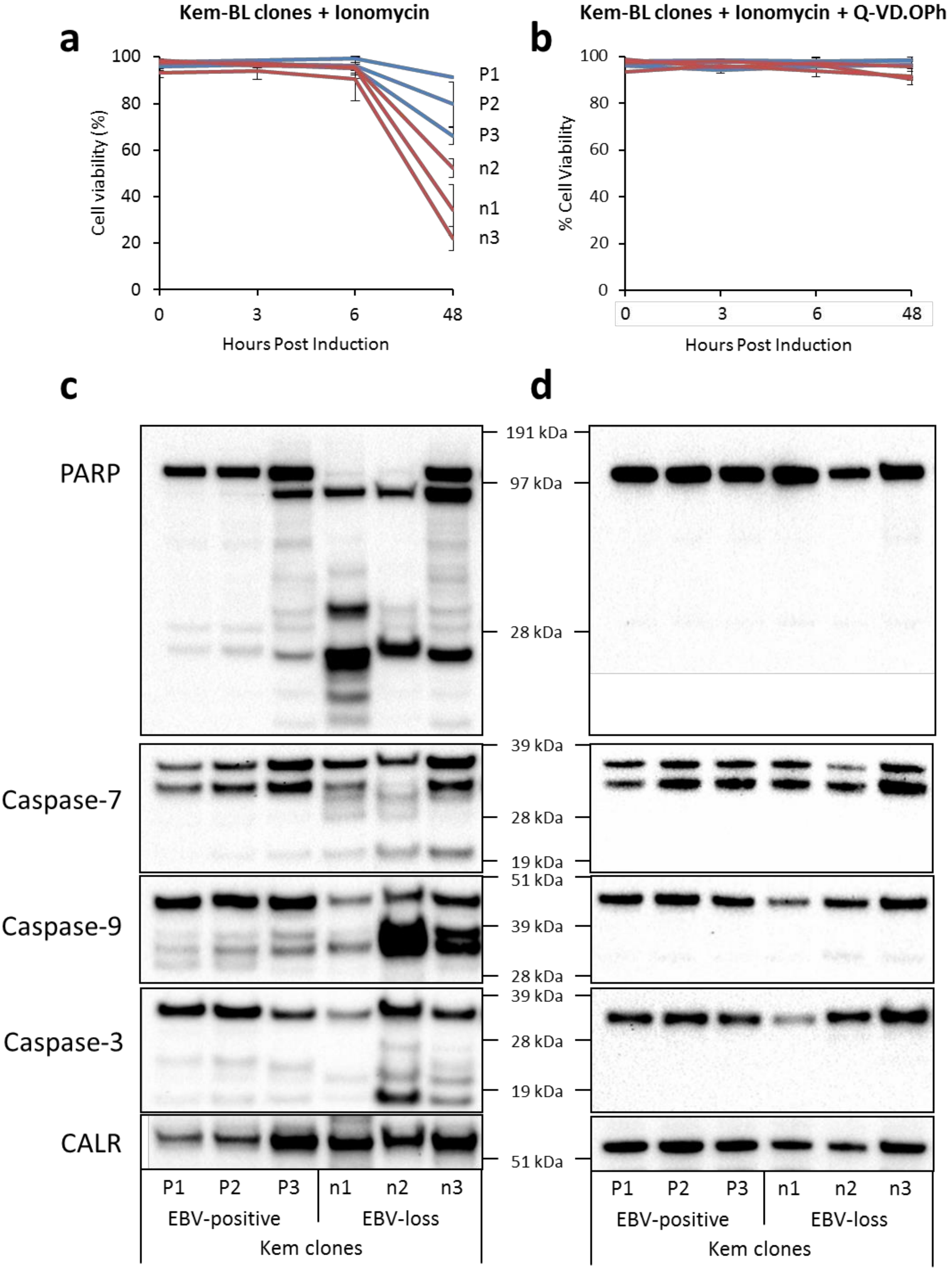

SI Figure 13. Viability and apoptosis-related protein expression in ionomycin-treated Kem-BL clones in the absence or presence of the pan-caspase inhibitor, Q-VD.OPh

(**a-b**) Viability of EBV-positive (blue, P1-P3) and EBV-loss (red, n1-n3) Kem-BL clones treated with 1 µg/ml ionomycin alone (**a**) 1 µg/ml ionomycin + 20 µM Q-VD.OPh (**b**) for 0, 3, 6 or 48 h .Viability was determined

by propidium iodide exclusion detected by flow cytometry. Data are mean and SD of three independent experiments. (**c-d**) Western blots showing full-length and cleaved forms of PARP and apoptosis-associated Caspases 3-, -7 and -9 as well as the loading control, calregulin (CALR) in EBV-positive (P1-P3) and EBV-loss (n1-n3) clones of Kem-BL treated with 1 µg/ml ionomycin for 48 h (**c**) or 1 µg/ml ionomycin + Q-VD.OPh for 48 h (**d**) Blots are representative of results obtained in three experiments.

SI Table 1. Summary of all pairwise comparisons between EBV-positive and EBV-loss clones of Kem-BL following treatment with ionomycin plus Q-VD.OPh

Gene	Fold-change	p-value	
**EBV-positive clones, 6 hrs vs EBV-positive clones, 0 hrs**			
BCL3	2.2	0.004	**
PMAIP1	2.1	0.015	*
TNF	-2.2	0.031	*
BIRC7	-5.9	0.0015	**
**EBV-positive clones, 48 hrs vs EBV-positive clones, 0 hrs**			
BBC3	3.4	0.018	*
CARD6	3.2	0.025	*
**EBV-loss clones, 6 hrs vs EBV-loss clones, 0 hrs**			
RELB	3.0	0.03	*
**EBV-loss clones, 48 hrs vs EBV-loss clones, 0 hrs**			
CFLAR	18.6	0.015	*
BIRC2	7.5	0.0016	**
CASP8	6.7	0.037	*
RELB	6.0	0.042	*
CASP8AP2	5.6	0.033	*
CASP7	5.1	0.0013	**
DEDD2	4.6	0.00016	***
BCL2L2	4.3	0.037	*
BCL2L13	4.0	0.043	*
TBK1	3.9	0.00087	***
IKBKG	3.4	0.043	*
APAF1	3.4	0.021	*
BIRC6	3.1	0.013	*
CHUK	3.0	0.0078	**
DAPK1	2.2	0.017	*
**EBV-loss clones, 48 hrs vs EBV-positive clones, 48 hrs**			
CASP8AP2	29.5	0.039	*
DEDD2	3.4	0.0094	**

Statistical significance was determined using a two-tailed Student's T-test, *** p <0.001, ** p <0.01, * p <0.05, ns is not significant.

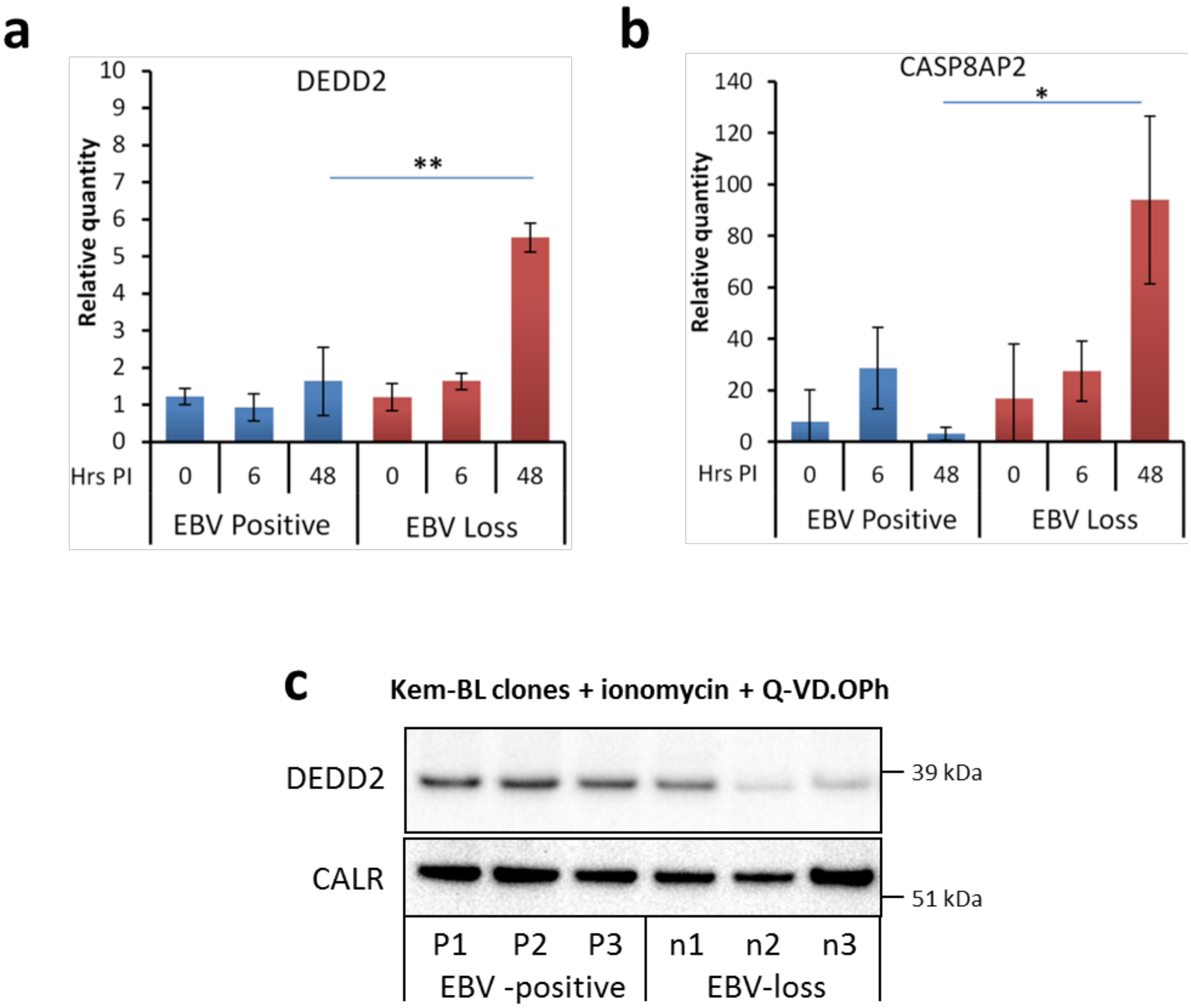

SI Figure 14. Validation of differential expression of DEDD2 and CASP8AP2 in EBV-loss versus EBV-positive Kem-BL clones after apoptosis induction

(**a**) DEDD2 and (**b**) CASP8AP2 transcription in EBV-positive and EBV-loss clones of Kem-BL after 0, 6 or 48 h treatment with ionomycin plus Q-VD.OPh, relative to EBV-positive clones at time 0. All data are from q-PCR carried out on three EBV-positive and three EBV-loss clones of Kem-BL, grouped by EBV status. All clones were treated with 1 µg/ml ionomycin and 20 µM Q-VD.OPh for 0, 6 or 48 h. Data are mean and SD of three independent experiments, each run in triplicate. Statistical significance was determined using a two-tailed Student's T-test, ** $p < 0.01$, * $p < 0.05$. (**c**) DEDD2 (also known as FLAME3) protein expression in EBV-positive (P1-P3) and EBV-loss (n1-n3) clones of Kem-BL treated with 1 µg/ml ionomycin plus 20 µM Q-VD.OPh for 48 h. Probing for calregulin (CALR) was included as a loading control. Images are representative from three independent experiments.

SI Table 2. Binding affinity profiles of BIM_S variants for cellular pro-survival BCL-2 family members and details of BIM_S variant design

		Binding to pro-survival BCL-2 homologues (nM)					Details of variant compared to BIM_S-wt
		BCL-2	BCL-XL	BCL-w	A1	MCL-1	
BIM_S variants	**wt**	2.6	4.6	4.3	4.3	2.4	Full-length human BIM_S, accession number: NP_001191035.1
	**BAD**	16	5.3	30	-	-	Amino acids (AAs) 51-76 replaced with AAs 103-126 of human BAD.
	**2a**	-	-	-	520	19	Two single AA mutations: L62A and F69A.
	**NOXA**	-	-	-	180	24	AAs 51-76 replaced with AAs 20-43 of human NOXA.
	**4e**	-	-	-	-	-	Four single AA mutations: I58E, L62E, I65E and F69E.

Affinity of the BIM_S variant proteins to the different pro-survival BCL-2 family members used in functional studies as measured by Biacore solution competition assays. A dash (-) denotes no binding. This information is collated from the publications in which these BIM_S variants were first described [7, 8].

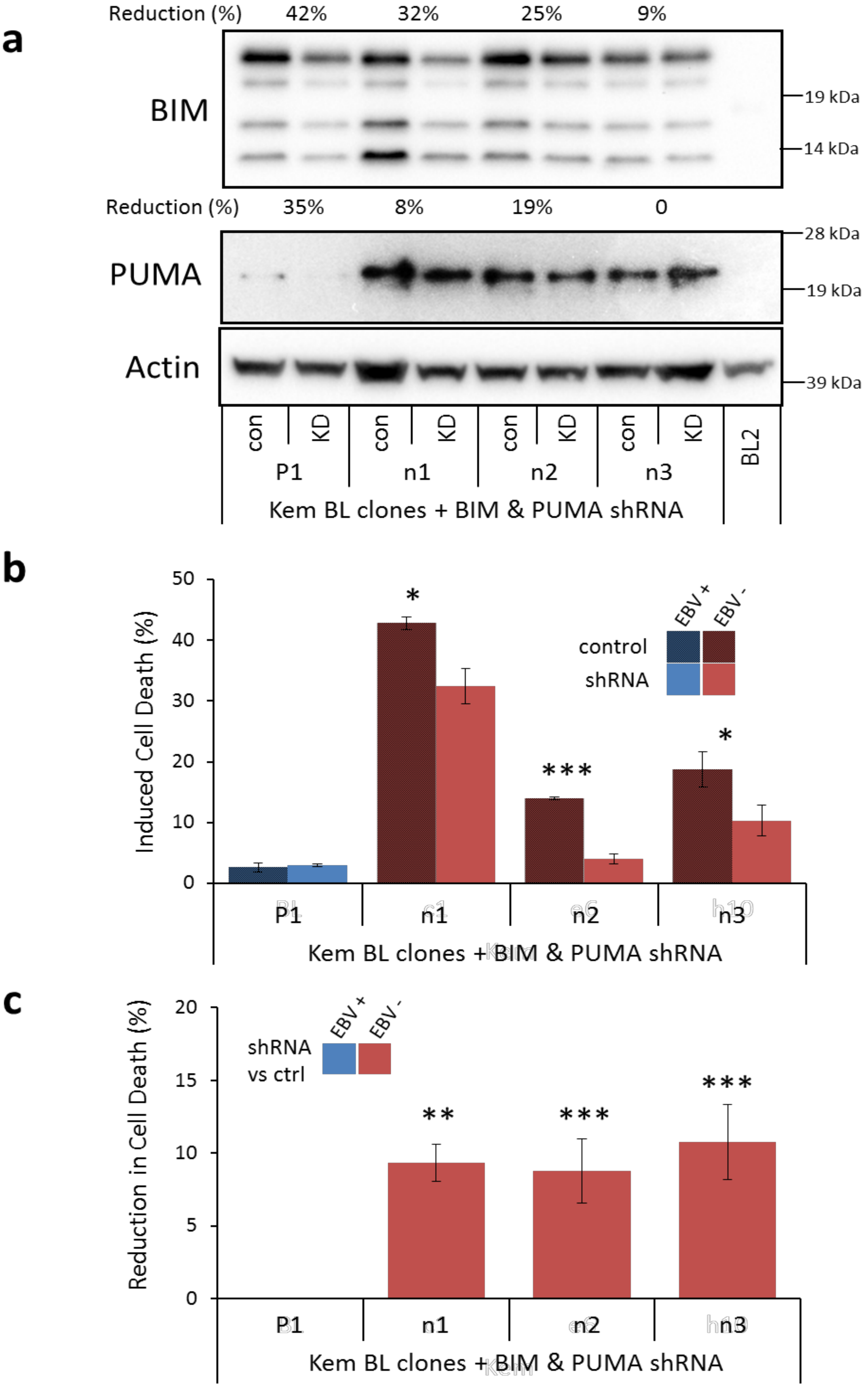

SI Figure 15. Effect of BIM and PUMA shRNA knockdown in clones of Kem-BL

(**a**) Western blotting for BIM and PUMA protein in EBV-positive (P1) and EBV-loss (n1-n3) clones of Kem-BL transduced with BIM and PUMA shRNA lentivirus constructs (KD) versus scrambled shRNA controls (con). BL2 was included as a control as it is negative for BIM and expresses very low levels of PUMA. (**b**) Total cell death in cells treated with ionomycin (1 µg/ml, 48 hrs) compared to untreated controls. (**c**) Reduction in cell death in BIM/PUMA KD cells compared to scrambled shRNA controls. Survival data are the mean and SD of three independent experiments each carried out in triplicate. The significance of any differences in survival between control and KD cells (middle panel) and EBV-positive and loss cells (lower panel) was determined using an unpaired, two-tailed, Student's T-test and are denoted as ***p <0.001, **p <0.01, * p<0.05.

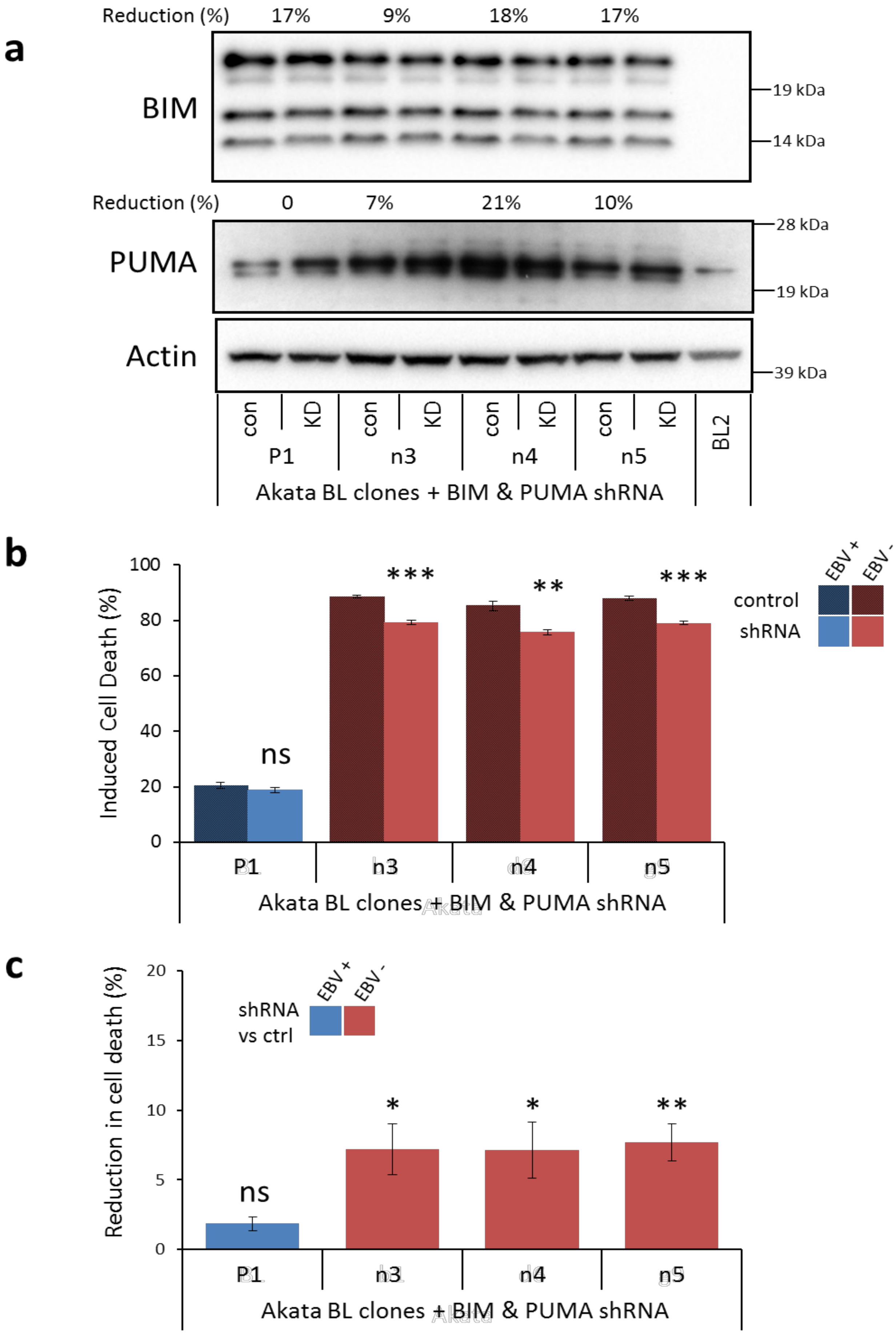

SI Figure 16. Effect of BIM and PUMA shRNA knockdown in clones of Akata-BL

(a) Western blotting for BIM and PUMA protein in EBV-positive (P1) and EBV-loss (n1-n3) clones of Akata-BL transduced with BIM and PUMA shRNA lentivirus constructs (KD) versus scrambled shRNA controls (con). BL2 was included as a control as it is negative for BIM and expresses very low levels of PUMA. (**b**) Total cell death in cells treated with ionomycin (1 µg/ml, 48 hrs) compared to untreated controls. (**c**) Reduction in cell death in BIM/PUMA KD cells compared to scrambled shRNA controls. Survival data are the mean and SD of three independent experiments each carried out in triplicate. The significance of any differences in survival between control and KD cells (middle panel) and EBV-positive and loss cells (lower panel) was determined using an unpaired, two-tailed, Student's T-test and are denoted as *** $p<0.001$, ** $p<0.01$, * $p<0.05$, ns $p>0.05$.

SUPPLEMENTARY METHODS

Plasmids, lentiviruses and recombinant EBVs

The recombinant Akata virus BAC construct is a derivative of the EBV strain isolated from the parental, EBV-positive Akata-BL cell line. It was developed by Prof Teru Kanda who kindly made it available for use in this study. The recombinant Akata EBV and the rEBV-containing cells used to produce infectious Ak-V stocks have been described in detail elsewhere [2]. The CpWp-KO B95.8-derived virus (B-V) is derived from the 2089 EBV BAC, but contains deletions to disrupt viral gene expression driven from the Wp and Cp promoters. The virus construct was made from the 0W BAC with an additional deletion spanning the entire Cp promoter, as previously described [3, 4].

FTrex-EBNA1-FLAG-UTG, contains the full-length *EBNA1* gene, which was subcloned from pRTS-CD2-EBNA1-FLAG [9] and inserted downstream of the Trex promoter. The sequence was then modified by site-directed mutagenesis to include an optimised Kozac sequence and a 3' polyA signal.

Genomic fragments encoding the two clusters of BART miRs were amplified from Raji-BL genomic DNA, cloned into the FUTG vector and then subcloned along with the Trex promoter into the FH1t-UTG vector as described elsewhere [10, 11]. Cluster 1 consisted of nucleotides 138997 to 140144 whilst Cluster 2 was comprised of nucleotides 145770 to 149022 of the EBV reference genome (AJ507799) using an identical strategy to that used by *Pratt et al* [12]. A miR-BART-5 only lentivirus was also constructed using the same strategy as *Choy et al* [13] except in this case the EBV genomic fragment was cloned into FH1-UTG where expression is driven from a dox-inducible, polIII, H1 promoter [10].

The EBER RNAs were expressed from the EKS10 plasmid, which contains ten concatamerised copies of the *EcoRI* J fragment of the EBV genome and expresses EBERs constitutively from their endogenous promoters and was developed by the Takada group [14]. The empty vector control was pcDNA3 and stable transfectants were generated by growing cells in selective medium containing G418 for several weeks.

BIM_S-variants are described in SI Table 2. Stable vector transduced cell lines were generated by subjecting lentivirus infected cells to two rounds of cell sorting for high GFP expression. We confirmed that BIM_S-variant proteins were expressed at comparable levels in each BL background by Western blotting before using these cells in functional assays. We did not use an additional BIM_S-PUMA construct as a mouse BIM-PUMA chimera has been shown to bind and inhibit pro-survival BCL-2 family members and therefore function as a tumour suppressor with equivalent efficiency to wt BIM in the *Eμ-Myc* lymphoma model [15, 16].

BIM and PUMA knockdown experiments were carried out using commercially available lentiviral shRNA constructs (α-BIM sc-29802, α-PUMA sc-37153 and scrambled control sc-108060 are all from Santa Cruz Biotechnology, Dallas, TX) and stable lines were generated by puromycin selection.

EBER ISH

EBER in situ hybridization (ISH) was performed by using an EBER ISH kit (Agilent, Santa Clara, CA) according to manufacturer's instructions as previously described [17]. EBER-positive cells were detected by flow cytometry using an Accuri C6 flow cytometer (BD). For each sample a minimum of 10,000 events were recorded and analysis was carried out using FlowJo software (Treestar). Gating was set to exclude dead cells, debris and doublets. Controls were: EBV-positive cells staining with EBER or negative control probe and EBV-negative cells stained with EBER probe.

Microarray gene expression analysis

In vitro transcribed, biotin-labelled, single-stranded cRNA from EBV-positive and loss BL clones was fragmented and hybridised to Affymetrix human genome U133 Plus 2.0 microarrays. Scanned images of microarry chips were analysed using GCOS (GeneChip Operating Software) from Affymetrix, Inc. (Santa Clara, California, USA) to generate CEL files. Probe level quantile normalisation [18] and robust multi-array analysis [19] on the raw CEL files was performed using the affy package of the Bioconductor project (http://www.biocondutor.org) and custom cdf HGU133Plus2_Hs_ENTREZG [20]. Differentially expressed genes were identified using limma [21] with tumour and EBV status as explanatory variables and with the criteria of absolute fold change > 2 and false discovery rate < 10%. Array data have been submitted to the GEO database under accession number GSE100458.

SUPPLEMENTARY REFERENCES

1. Ressing, M.E. *et al.* Impaired transporter associated with antigen processing-dependent peptide transport during productive EBV infection. *J Immunol* **174**, 6829-6838 (2005).
2. Kanda, T., Yajima, M., Ahsan, N., Tanaka, M. & Takada, K. Production of high-titer Epstein-Barr virus recombinants derived from Akata cells by using a bacterial artificial chromosome system. *J Virol* **78**, 7004-7015 (2004).
3. Tierney, R.J., Kao, K.Y., Nagra, J.K. & Rickinson, A.B. Epstein-Barr virus BamHI W repeat number limits EBNA2/EBNA-LP coexpression in newly infected B cells and the efficiency of B-cell transformation: a rationale for the multiple W repeats in wild-type virus strains. *J Virol* **85**, 12362-12375 (2011).
4. Tierney, R.J., Nagra, J., Rowe, M., Bell, A.I. & Rickinson, A.B. The Epstein-Barr virus BamHI C promoter is not essential for B cell immortalization in vitro, but it greatly enhances B cell growth transformation. *J Virol* **89**, 2483-2493 (2015).
5. Dave, S.S. *et al.* Molecular Diagnosis of Burkitt's Lymphoma. *New England Journal of Medicine* **354**, 2431-2442 (2006).
6. Hummel, M. *et al.* A biologic definition of Burkitt's lymphoma from transcriptional and genomic profiling. *N Engl J Med* **354**, 2419-2430 (2006).
7. Lee, E.F. *et al.* A novel BH3 ligand that selectively targets Mcl-1 reveals that apoptosis can proceed without Mcl-1 degradation. *J Cell Biol* **180**, 341-355 (2008).
8. Chen, L. *et al.* Differential targeting of prosurvival Bcl-2 proteins by their BH3-only ligands allows complementary apoptotic function. *Mol Cell* **17**, 393-403 (2005).
9. Leung, C.S., Haigh, T.A., Mackay, L.K., Rickinson, A.B. & Taylor, G.S. Nuclear location of an endogenously expressed antigen, EBNA1, restricts access to macroautophagy and the range of CD4 epitope display. *Proc Natl Acad Sci U S A* **107**, 2165-2170 (2010).
10. Herold, M.J., van den Brandt, J., Seibler, J. & Reichardt, H.M. Inducible and reversible gene silencing by stable integration of an shRNA-encoding lentivirus in transgenic rats. *Proc Natl Acad Sci U S A* **105**, 18507-18512 (2008).
11. Aubrey, B.J. *et al.* An inducible lentiviral guide RNA platform enables the identification of tumor-essential genes and tumor-promoting mutations in vivo. *Cell Rep* **10**, 1422-1432 (2015).
12. Pratt, Z.L., Kuzembayeva, M., Sengupta, S. & Sugden, B. The microRNAs of Epstein-Barr Virus are expressed at dramatically differing levels among cell lines. *Virology* **386**, 387-397 (2009).
13. Choy, E.Y. *et al.* An Epstein-Barr virus-encoded microRNA targets PUMA to promote host cell survival. *J Exp Med* **205**, 2551-2560 (2008).
14. Komano, J., Maruo, S., Kurozumi, K., Oda, T. & Takada, K. Oncogenic role of Epstein-Barr virus-encoded RNAs in Burkitt's lymphoma cell line Akata. *J Virol* **73**, 9827-9831 (1999).
15. Merino, D., Strasser, A. & Bouillet, P. Bim must be able to engage all pro-survival Bcl-2 family members for efficient tumor suppression. *Oncogene* **31**, 3392-3396 (2012).

16. Merino, D. *et al.* The role of BH3-only protein Bim extends beyond inhibiting Bcl-2-like prosurvival proteins. *J Cell Biol* **186**, 355-362 (2009).
17. Shannon-Lowe, C. *et al.* Features distinguishing Epstein-Barr virus infections of epithelial cells and B cells: viral genome expression, genome maintenance, and genome amplification. *J Virol* **83**, 7749-7760 (2009).
18. Bolstad, B.M., Irizarry, R.A., Astrand, M. & Speed, T.P. A comparison of normalization methods for high density oligonucleotide array data based on variance and bias. *Bioinformatics* **19**, 185-193 (2003).
19. Irizarry, R.A. *et al.* Summaries of Affymetrix GeneChip probe level data. *Nucleic Acids Res* **31**, e15 (2003).
20. Dai, M. *et al.* Evolving gene/transcript definitions significantly alter the interpretation of GeneChip data. *Nucleic Acids Res* **33**, e175 (2005).
21. Smyth, G.K. Linear models and empirical bayes methods for assessing differential expression in microarray experiments. *Stat Appl Genet Mol Biol* **3**, Article3 (2004).
